# Prior Biological Knowledge Improves Genomic Prediction of Growth-Related Traits in *Arabidopsis thaliana*

**DOI:** 10.3389/fgene.2020.609117

**Published:** 2021-01-20

**Authors:** Muhammad Farooq, Aalt D. J. van Dijk, Harm Nijveen, Mark G. M. Aarts, Willem Kruijer, Thu-Phuong Nguyen, Shahid Mansoor, Dick de Ridder

**Affiliations:** ^1^Bioinformatics Group, Wageningen University, Wageningen, Netherlands; ^2^Molecular Virology and Gene Silencing Lab, Agricultural Biotechnology Division, National Institute for Biotechnology and Genetic Engineering (NIBGE), Punjab, Pakistan; ^3^Biometris, Wageningen University, Wageningen, Netherlands; ^4^Laboratory of Genetics, Wageningen University, Wageningen, Netherlands

**Keywords:** genomic prediction (GP), photosynthesis, phenomics data analysis, *Arabidopsis thaliana* (Arabidopsis), GBLUP, GFBLUP

## Abstract

Prediction of growth-related complex traits is highly important for crop breeding. Photosynthesis efficiency and biomass are direct indicators of overall plant performance and therefore even minor improvements in these traits can result in significant breeding gains. Crop breeding for complex traits has been revolutionized by technological developments in genomics and phenomics. Capitalizing on the growing availability of genomics data, genome-wide marker-based prediction models allow for efficient selection of the best parents for the next generation without the need for phenotypic information. Until now such models mostly predict the phenotype directly from the genotype and fail to make use of relevant biological knowledge. It is an open question to what extent the use of such biological knowledge is beneficial for improving genomic prediction accuracy and reliability. In this study, we explored the use of publicly available biological information for genomic prediction of photosynthetic light use efficiency (Φ_*PSII*_) and projected leaf area (PLA) in *Arabidopsis thaliana*. To explore the use of various types of knowledge, we mapped genomic polymorphisms to Gene Ontology (GO) terms and transcriptomics-based gene clusters, and applied these in a Genomic Feature Best Linear Unbiased Predictor (GFBLUP) model, which is an extension to the traditional Genomic BLUP (GBLUP) benchmark. Our results suggest that incorporation of prior biological knowledge can improve genomic prediction accuracy for both Φ_*PSII*_ and PLA. The improvement achieved depends on the trait, type of knowledge and trait heritability. Moreover, transcriptomics offers complementary evidence to the Gene Ontology for improvement when used to define functional groups of genes. In conclusion, prior knowledge about trait-specific groups of genes can be directly translated into improved genomic prediction.

## Introduction

Due to breakthroughs in DNA sequencing technology over the past decade, high-throughput genotyping is now a routine practice in plant breeding (Rimbert et al., 2018). Phenotyping is undergoing a similar revolution: large phenomics facilities are being developed that can rapidly score large germplasm collections of plants in a range of different environments (Flood et al., 2016; Crain et al., 2018). These technological developments have made it possible to acquire datasets describing genotypes and phenotypes for large numbers of individuals at an extended temporal scale. Despite recent advances in phenomics it is still more expensive and laborious than genotyping. To make the most use of phenomic datasets, Genomic Selection (GS) based breeding programs aim to predict unobserved phenotypes of individuals based on genotypes alone. This has the twofold benefit of reducing breeding costs and speeding up breeding programs as plants can be genotyped in the seedling stage and selected accordingly, thus negating the need to grow large populations to maturity and scoring them all to obtain breeding values based on phenotypes. GS usually models the unobserved phenotypes as additive effects of all genetic markers (total additive genomic value or breeding value) in the test population using a genomic prediction (GP) model. This GP model is based on a reference population which has both been genotyped and phenotyped for the trait(s) of interest (Meuwissen et al., 2001). The performance of GP depends on many factors, including genetic architecture, reference population size and structure and heritability (Karaman et al., 2016). However, GP accuracy, usually defined as the correlation (Pearson's *r*) between observed phenotypes and predicted breeding values, is generally lower for complex traits than for simpler ones (Morgante, 2018). This is because such traits are affected by many loci with small to moderate effects, along with non-additive genetic (dominance, epistasis) and genotype-by-environment (GxE) interactions (Falconer and Mackay, 1996). Incorporating epistasis into GP models has been reported to improve performance in selfing plant species but may not work for outcrossing species; therefore, additive GP models are still the primary choice (Jiang and Reif, 2015).

In GP models, each individual's genetic or breeding value is modeled as the sum of additive marker effects. Despite advancements in phenomics, phenotyping data is still usually only available for a few traits of several hundreds of individuals (*n*), compared to millions of genetic markers (*p*). GP models tackle this curse of dimensionality (*p* > *n*) by regularization (Meuwissen et al., 2001). When marker effects are fixed, this comes in the form of a penalty term added to the log-likelihood, as in LASSO or ridge regression. More frequently, marker effects are considered random, and regularization is achieved through prior distributions on the marker effects. The variance in these priors is directly related to the heritability, and can be estimated either using REML, or a fully Bayesian approach. In the classical GBLUP-approach, a single normal distribution with equal variance is assumed for all marker effects (Vanraden, 2008). More recently, mixture distributions have been considered (Moser et al., 2015). The prior could e.g., be a mixture of Gaussian distributions with large and small variances, and a point mass at zero, allowing a marker to have respectively, large or small effects, or no effect at all (Macleod et al., 2016). Moreover, restrictions on the shape of the probability distribution, usually Gaussian, can be relaxed (e.g., *t*-distribution) to accommodate genetic architectures having a larger number of high to moderate effect sizes (Gianola, 2013) or another suitable distribution can be exploited instead. In spite of these refinements, it is usually impossible to find the true causal variants when *p* > *n*, which may lead to suboptimal prediction. Therefore, several authors suggested that *a priori* available biological knowledge may be incorporated in GP models, prioritizing likely causal markers, and ultimately improving prediction accuracy (Edwards et al., 2016; Ehsani et al., 2016; Wang et al., 2018).

Two types of biological knowledge have been considered in the literature: first, knowledge on biological properties of genes and their associated markers and second, knowledge in the form of secondary phenotypes. The latter typically concerns -omics data, and is modeled using additional relatedness matrices (Guo et al., 2016; Morgante, 2018; Azodi et al., 2020) or penalized selection indices (Lopez-Cruz et al., 2020). Although such -omics data can in principle be generated for the GP reference population, the use of more general publicly available information is often more feasible and cost-effective. We therefore focus on biological properties of genes and markers, such as Gene Ontology (GO) and post-GWAS QTL information. The GO provides a structured resource of functional classes of gene products based on orthology, represented into three biological domains, i.e., molecular function, cellular component and biological process (Ashburner et al., 2000). Similar functional groupings can be achieved from transcriptomic experiments based on the assumption that functionally related genes are expressed together. These clusters of co-expressed genes may be enriched in multiple GO terms or pathways. Such information can be incorporated by allowing the GP model to put more weight on either certain individual markers (Legarra and Ducrocq, 2012; Macleod et al., 2016) or groups of markers (Edwards et al., 2016). Various modeling approaches have been proposed to enable use of such data (Zhang et al., 2010; Speed and Balding, 2014; Edwards et al., 2016; Ehsani et al., 2016; Guo et al., 2016; Fragomeni et al., 2017). Here we use the Genomic Feature Best Linear Unbiased Predictor (GFBLUP) approach proposed by Edwards et al., 2016. GFBLUP extends GBLUP by partitioning the total genomic variance into two sub-components to weigh different genomic regions differently. This allows incorporating prior biological knowledge about groups of variants by treating each region as a separate random genetic effect with different variance. Subsequently, researchers applied this approach to various traits (Sarup et al., 2016; Fang et al., 2017; Rohde et al., 2017; Gebreyesus et al., 2019). While prior biological knowledge has thus been used to improve GP accuracy, the question remains what type of knowledge is most useful and how much the genetic architecture impacts the potential for improvement of particular traits.

In this study, we investigate improvement in GP performance using two sources of publicly available biological knowledge, i.e., Gene Ontology (GO) and clusters of co-expressed genes (COEX). This information was incorporated using the GFBLUP modeling approach, grouping markers in genes according to either their predicted function or co-expression, respectively. As complex traits of study, we focused on photosynthetic light use efficiency of photosystem II (Φ_*PSII*_) and projected leaf area (PLA) in *Arabidopsis thaliana*. Both of these traits are related, in the sense that the Φ_*PSII*_ directly illustrates the photosynthetic light use efficiency and can capture the most immediate physiological and regulatory response to varying irradiance levels (Van Rooijen et al., 2015), whereas growth in PLA is the net outcome of unit leaf photosynthetic capacity over time (Weraduwage et al., 2015; Liu et al., 2020).

## Results

### Genomic Prediction of Complex Growth Related Traits

**Figure 1:**
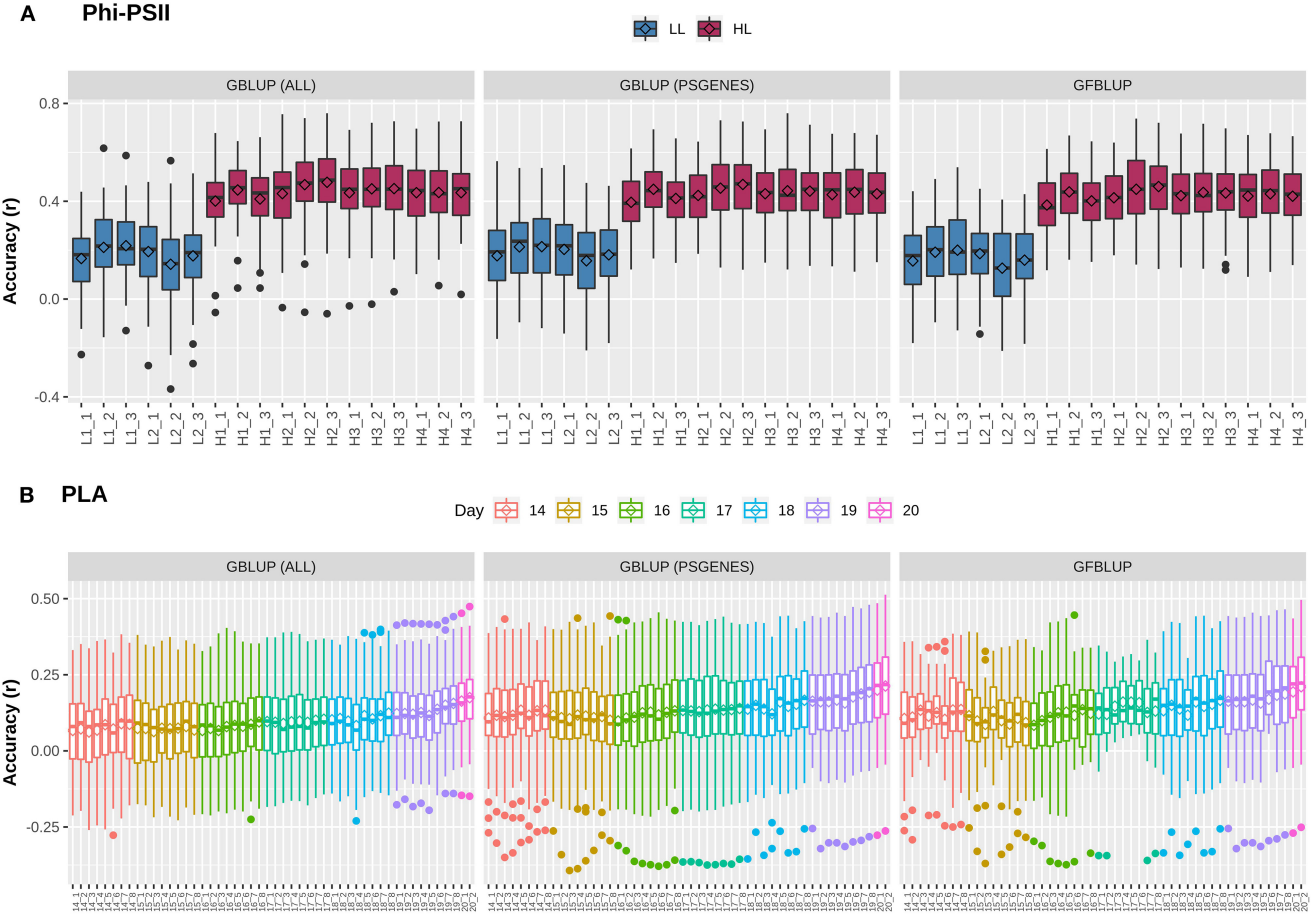
Genomic prediction accuracy for photosynthetic light efficiency and projected leaf area. Cross-validation based assessment of prediction accuracy as Pearson's *r* between true and predicted values using three models: (i) GBLUP using all genomic markers (ALL), (ii) GBLUP using only pre-selected photosynthesis related genic markers (*PSGENES*) and (iii) GFBLUP using *PSGENES* as a genomic feature in two genomic components. **(A)** Accuracy for Φ_*PSII*_ for two days (6 time points) under low light irradiance levels (LL) and four days (12 time points) under high light irradiance (HL). **(B)** Accuracy for PLA measured 8 times per day from day 14 after sowing to day 20, where day 20 has only two measurements.

Previously, Van Rooijen et al. (2017) conducted a GWAS on *A. thaliana* photosynthesis. In particular, they measured the light use efficiency of photosystem II electron transport (Φ_*PSII*_) for 344 accessions of the Arabidopsis HapMap population, switching from low light (100 μmol m^−2^ s^−1^) to high light (550 μmol m^−2^ s^−1^) irradiance at the onset of day 25. In total, they took 6 measurements before and 12 after applying light stress to identify potential QTLs during acclimation to high light. As we intend to use this population to explore the utility of biological knowledge in genomic prediction, we combined projected leaf area (PLA), another indicator of plant growth, with Φ_*PSII*_. We first assessed whether GP works with reasonable performance for these complex traits. For this purpose, a classical Genomic Best Linear Unbiased Prediction (GBLUP) model was constructed to assess how well the infinitesimal modeling assumptions fit and to calculate markers-based heritability. In this model (Equation 2), all marker effects are treated as arising from a single normal distribution $N\left(0,\text{G}\sigma_{\text{g}}^{2}\right)$ having one random genetic component, to regress each individual phenotype measurement over all markers simultaneously. At low light (LL) levels, mean prediction accuracy for Φ_*PSII*_ is lower (Pearson's *r* between predicted and observed phenotypic values ranging from 0.16 ± 0.02 to 0.22 ± 0.01) than at high light (HL, Pearson's *r* ranging from 0.40 ± 0.01 to 0.48 ± 0.01), as shown in Figure 1A. Prediction accuracy for PLA (Figure 1B) ranges from 0.06 ± 0.01 to 0.17 ± 0.01 and rises with the increase in plant size and simultaneously decreases with increase in phenotypic coefficient of variation. Genomic heritability ($h_{GBLUP}^{2}$) for Φ_*PSII*_ ranged from 0.08 to 0.13 under LL and 0.56 to 0.87 under HL, and 0.05 to 0.17 for PLA (Supplementary Figure 1). Differences in prediction accuracy for Φ_*PSII*_ between LL and HL are in line with differences in genomic heritability, in accordance with the observation that genomic prediction accuracy is generally positively correlated with heritabilities (Hayes et al., 2009). Moreover, for ~1.2% of the GBLUP models for PLA, $h_{GBLUP}^{2}$ was zero because of undetermined genomic variance, whereas for Φ_*PSII*_ ~7% of genomic variances were estimated to be 100% $\left(h_{GBLUP}^{2}=1\right)$), which is clearly an over-estimation (Supplementary Figure 2). As reported by Kruijer et al., 2015, it was expected (based on 5000 simulated traits) that ~10-15% of GBLUP models could have variance components that cannot be estimated for this population, so we discarded these models from our analysis.

An extension of GBLUP is MultiBLUP (Speed and Balding, 2014), using multiple random genetic components in the model (Equation 4), thus allowing differential weighting of groups of genomic markers, each having a separate kinship matrix derived from that group. We applied MultiBLUP using adjacent overlapping chromosomal partitions of 10 kb (yielding best performance when testing window sizes of 1 to 100 kb) to check if multiple kinship matrices or genomic variance decomposition improve prediction. The results (Supplementary Figure 3) indicate that performance was close to that of GBLUP and could not be improved further. This could be because most models ended up with only one background kinship matrix during cross-validation and many of these genomic regions did not meet the significance threshold (*p*_Bonferroni_ < 0.05) during association testing. In summary, these results show that predictive performance for these complex traits is low and there may be room for improvement by incorporating prior biological knowledge, decomposing the total genomic variance into biologically relevant subsets.

### High-Level Biological Knowledge Does Not Necessarily Improve Genomic Prediction

The next question is whether predictive performance can be improved by using only markers residing within genes that are known to be linked to the traits of interest. The idea comes from previous studies, in which a subset of markers was associated to biological relevant genes and achieved a genomic value similar to the total genomic value achieved when using all SNPs (Vanraden et al., 2017; Li et al., 2018). Here, we selected 7,242 photosynthesis related genes, referred to as *PSGENES* in the text, from public repositories (see M&M) and constructed a GBLUP model based only on these. The Genomic Relationship Matrix (GRM) was constructed from all markers within the ORFs of *PSGENES*, leaving ~17% of the total genotyped markers after filtering. Interestingly, the models performed equally well (Figure 1) as the GBLUP based on all markers for both traits, with a slight improvement in predictive ability for PLA (max. ~6% increase in accuracy). Subsequently, to assess whether this pre-selected subset of markers can improve results if they are weighted differently than the rest of markers, we constructed another model using the GFBLUP modeling approach (Edwards et al., 2016) (Equation 3) having two genomic components. In this model, the markers within *PSGENES* were treated as one genomic component and the remaining markers as a second genomic component. Again, this model showed similar predictive performance as GBLUP, with some reduction in variability for PLA, but could not improve the accuracy further (Figure 1). From this, we conclude that prior biological knowledge-based selection of functionally relevant genes is potentially useful, but an optimal grouping may be important to improve GP further.

### More Fine-Grained Biological Knowledge Is Useful for Improving Genomic Prediction

To assess whether prior information from publicly available resources can help improve GP performance, we tested grouping of genes based on Gene Ontology (GO) terms and previously reported clusters of co-expressed genes (COEX) of *Arabidopsis thaliana* in multiple tissues and developmental stages (Movahedi et al., 2011). Each of the three GO sub-ontologies, Biological Process (BP), Molecular Function (MF) and Cellular Component (CC), was used. The corresponding groups of markers in a GO or COEX group, called a genomic feature (GF), were used in GFBLUP (Equation 3) using a separate model for each feature with two genomic components, i.e., one with markers from the GF and the other with the remaining markers (rGF). The predictive performance was compared to that of the GBLUP benchmark using all markers with identical sets of 8-fold cross-validation test populations. Each group of markers based on GO or COEX was treated as a separate random effect in its respective GFBLUP model for which its contribution to the total genomic variance was calculated (see M&M). For each GF, the effects of all corresponding markers were assumed to follow a normal distribution with equal variance, but different from the remaining markers; that is, the markers in the GF are differentially weighted and prioritized from the rest.

In total, 7,297 GO terms and 12,419 disjoint COEX gene groups were linked to at least one marker. The total number of genes ranged between 1 and 24,998 for the GO features and between 1 and 3,384 for the COEX groups (Supplementary Figure 4, Supplementary Table 4); the number of markers ranged between 0 and 109,723 for the GO features and 4 and 19,621 for the COEX groups. Due to the hierarchical GO structure, the 95^th^ percentile of the total number of genes within GO features was lower (496) as compared to COEX (2,466). Note that both GO and COEX groups may overlap, i.e., a gene can be in multiple functionally related GO/COEX groups. In the following results, the improvement in genomic prediction has been quantified in terms of percent gain in accuracy compared to the GBLUP benchmark, GFBLUP model's goodness of fit measured using likelihood ratio test (LR), and genomic heritability ($h_{GBLUP}^{2}$) and proportion of genomic heritability explained by a genomic feature ($h_{f}^{2}$).

### GO Informed Prediction

7,297 GO terms were tested with repeated 8-fold cross-validation at multiple measurements of a trait, leading to a total of ~10 million GFBLUP model accuracies for Φ_*PSII*_ and ~29 million for PLA (Supplementary Figure 5). The models for which variance was apparently over-estimated ($h_{f}^{2}>0.99$) or undetermined ($h_{f}^{2}<0.01$) were not considered for subsequent analysis. This was the case for ~50% of the models for both traits, indicating that only selected biological groups are potentially helpful.

**Figure 2:**
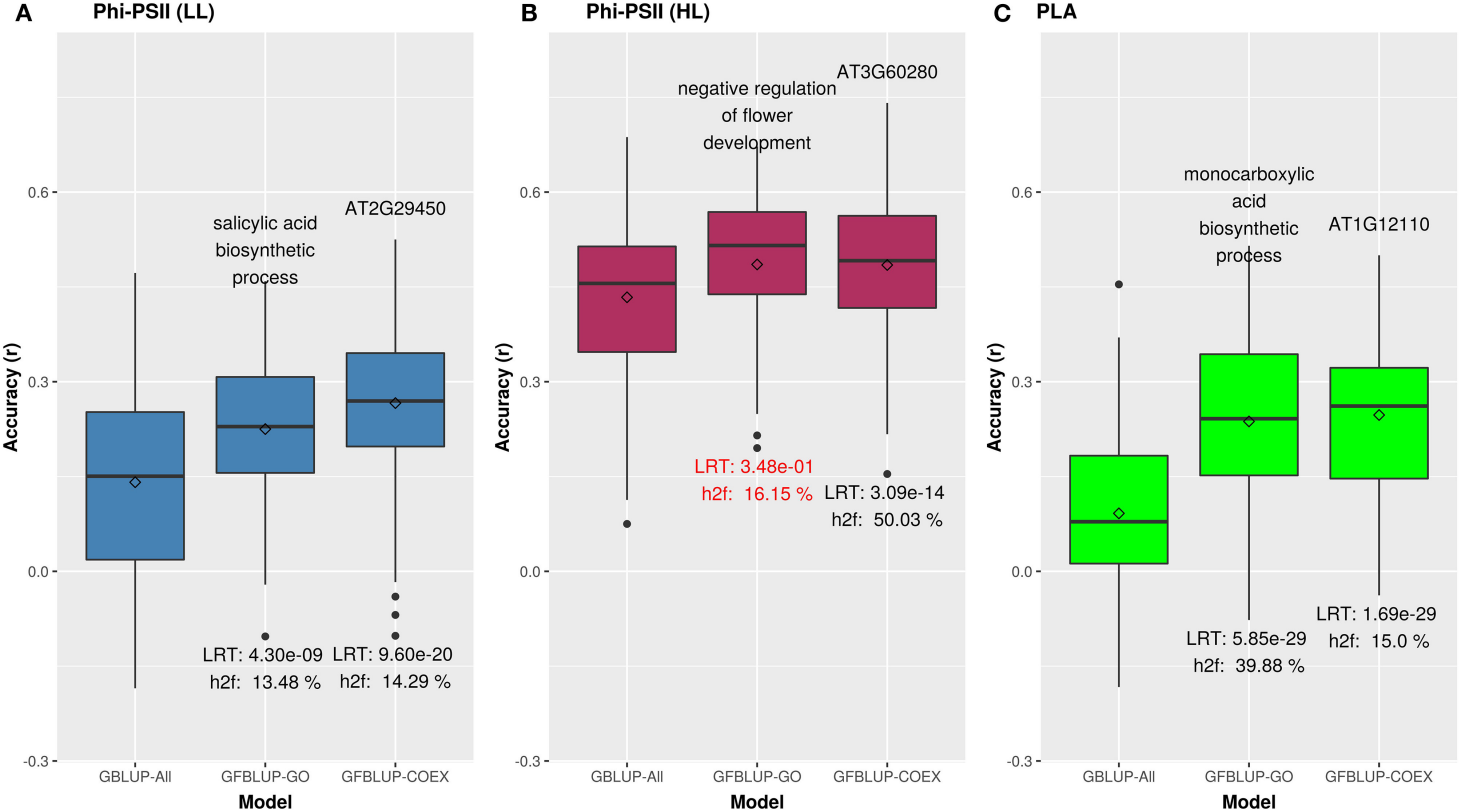
Biological priors can help improve genomic prediction accuracy for growth related traits. Prediction accuracy of the best overall GFBLUP models using Gene Ontology (GO) and co-expression (COEX) gene groups, compared to the GBLUP benchmark (without prior biological knowledge). Since the GBLUP model was evaluated for each measurement point, so the GBLUP here is shown for the corresponding time point where improvement by GFBLUP was observed. Accuracy was calculated as Pearson's *r* between true vs. predicted values. The GBLUP-ALL model uses all markers in GBLUP; GFBLUP-GO and GFBLUP-COEX models use the top GO terms and COEX (see text for details). **(A)** Accuracy for Φ_*PSII*_ under low light irradiance levels (LL). **(B)** Accuracy for φ_PSII_ under high light irradiance (HL). Here, despite showing some improvement, the GFBLUP-GO model did not pass all of our model evaluation criteria (see Model Performance Evaluation). **(C)** Accuracy for PLA.

**Figure 3:**
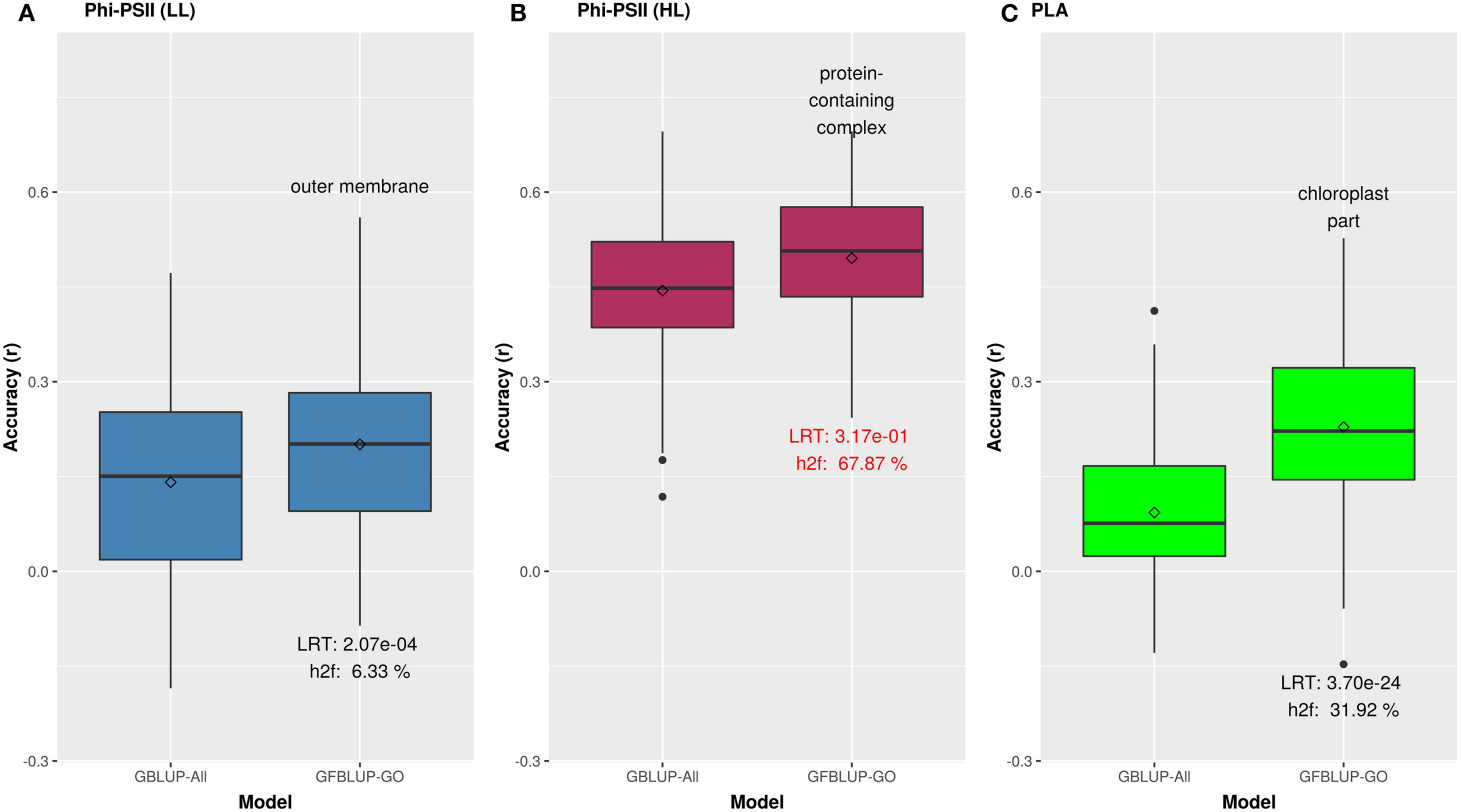
Biological priors based on top GO cellular components improving genomic prediction accuracy for growth related traits. Prediction accuracy of the best GFBLUP models using Gene Ontology (GO) cellular components gene groups, compared to the GBLUP benchmark (without prior biological knowledge). The accuracy of benchmark model may differ within corresponding figures of Figures 2, 3, because it is calculated from the corresponding time point, where improvement by GFBLUP was observed. Accuracy was calculated as Pearson correlation between true vs. predicted values. The GBLUP-ALL model uses all markers in GBLUP; GFBLUP-GO models use the top GO cellular component terms mentioned in the text above. The text in the bottom of boxplots shows the likelihood ratio test *p*-value (LRT) and proportion of genomic heritability explained (*h*${}_{\text{f}}^{2}$) by corresponding GO model. **(A)** Accuracy for Φ_*PSII*_ under low light irradiance levels (LL). **(B)** Accuracy for Φ_*PSII*_ under high light irradiance (HL). Similar to Figure 2, the GFBLUP-GO model did not pass all of our model evaluation criteria (see Model Performance Evaluation), though showing some improvement. **(C)** Accuracy for PLA.

We initially analyzed the highest gain in prediction performance obtained by any GO term at any time point. For Φ_*PSII*_, “salicylic acid biosynthesis” (BP) provided the highest increase in accuracy (~60%), for Φ_*PSII*_ measurements under low light on the second day (Figure 2, Supplementary Table 2A). For the GO sub-ontologies CC and MF, “organelle outer membrane” and “phosphatase activity,” respectively yielded highest gains in these categories under low light (~43 and 37%, respectively; Supplementary Table 2A). None of the GO terms yielded a significant improvement after high light stress; however, some GO terms, e.g., “protein containing complex” yielded an increase in accuracy higher than the benchmark but not passing our model evaluation criteria wholly (Figure 3). For PLA, the largest improvement (~197%) was obtained by the biological process “monocarboxylic acid biosynthesis” (Figure 2, Supplementary Table 2B). The best performing MF and CC terms for PLA were “exopeptidase activity” and “chloroplast part” (~185 and ~178%, respectively; Figure 3, Supplementary Table 2B). Interestingly, these best CC terms for both traits are directly related to photosynthesis, which lends credibility to the usefulness of the GO terms to capture relevant prior biological knowledge.

**Figure 4:**
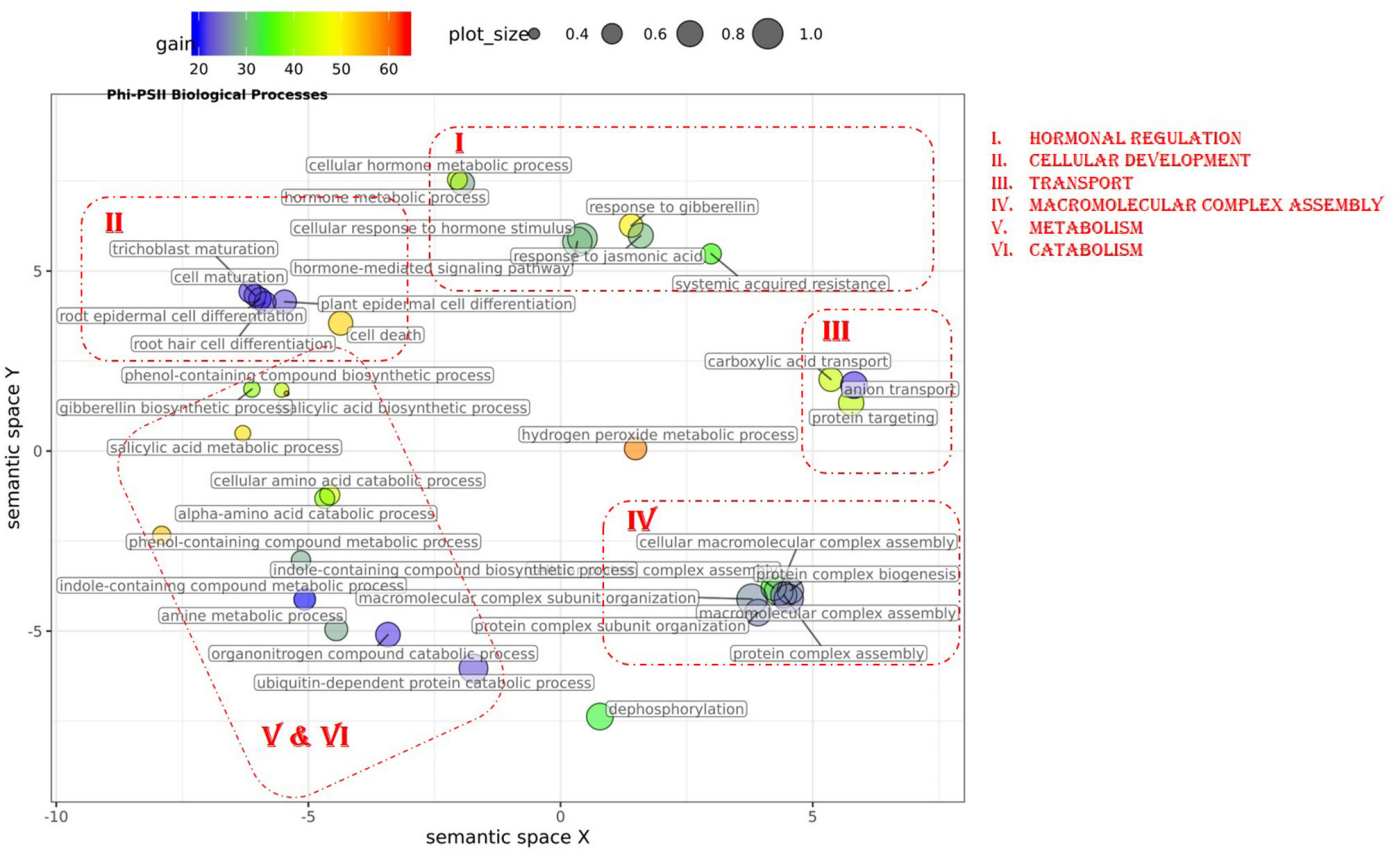
Semantic clustering of GO informed prediction for Φ_*PSII*_. Multidimensional scaling (MDS) plot of the representative subset (i.e., terms remaining after the redundancy reduction) of biological process GO terms informative for Φ_*PSII*_. Semantically similar GO terms are clustered based on the “*SimRel”* semantic similarity measure using *Revigo*. Dot size is proportional to the number of genes annotated with the GO term, such that more general GO terms have larger circles. The *x* and *y* coordinates indicate relative cluster distances in 2 dimensions. The %gain of a particular GO term is indicated by the circle color.

In total, 43 GO terms (BP:34, CC:6, MF:3) were potentially informative (i.e., Wilcoxon–Mann–Whitney test *p*-values < 0.05, without multiple testing correction), showing a tendency to improve Φ_*PSII*_ traits and yielding a significant increase in GFBLUP model accuracy (Supplementary Figures 6A, 7, Supplementary Table 2A) compared to GBLUP. The overall gain in accuracy for these informative GO features ranged between 23 and 60%. The GO terms' hierarchical redundancy was removed using GO trimming (Jantzen et al., 2011) and the remaining 40 informative terms fell broadly into six biological clusters (Figure 4, Supplementary Figure 9): (i) hormonal regulation; (ii) cellular development; (iii) transport; (iv) metabolism; (v) catabolism and (vi) macromolecular complex assembly, organization, and biogenesis. The cellular component terms were semantically clustered into organellar membranes and photosynthesis machinery sub-compartments, whereas molecular function terms were related to transmembrane transport and phosphatase activities.

**Figure 5:**
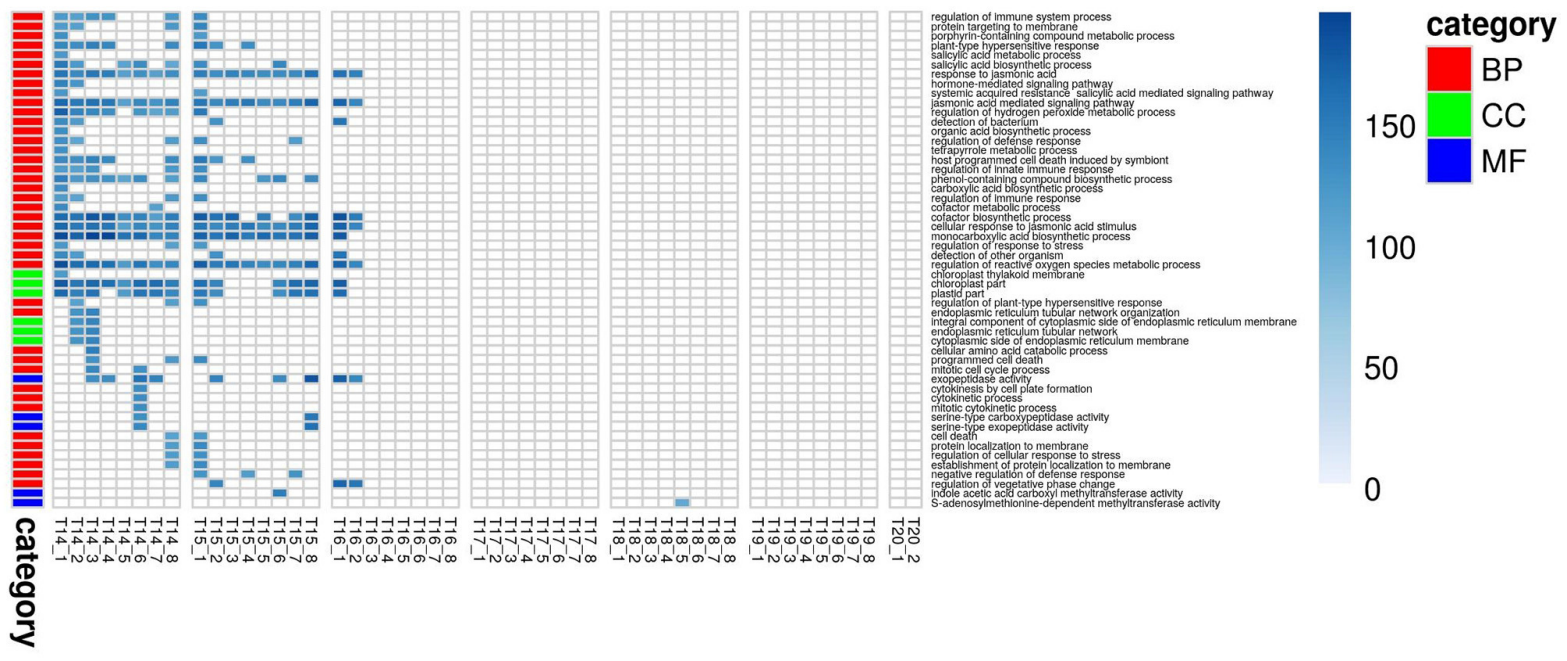
Improvement in genomic predictive performance using GO for PLA. All GO terms that significantly improve GFBLUP models for PLA with %gain in accuracy (*r*) over GBLUP. Each GO term has a separate model for individual measurements indicated as T{day}_{Number of measurement}. The color bar identifies the GO terms as Biological Process (BP), Cellular Component (CC) and Molecular Function (MF).

**Figure 6:**
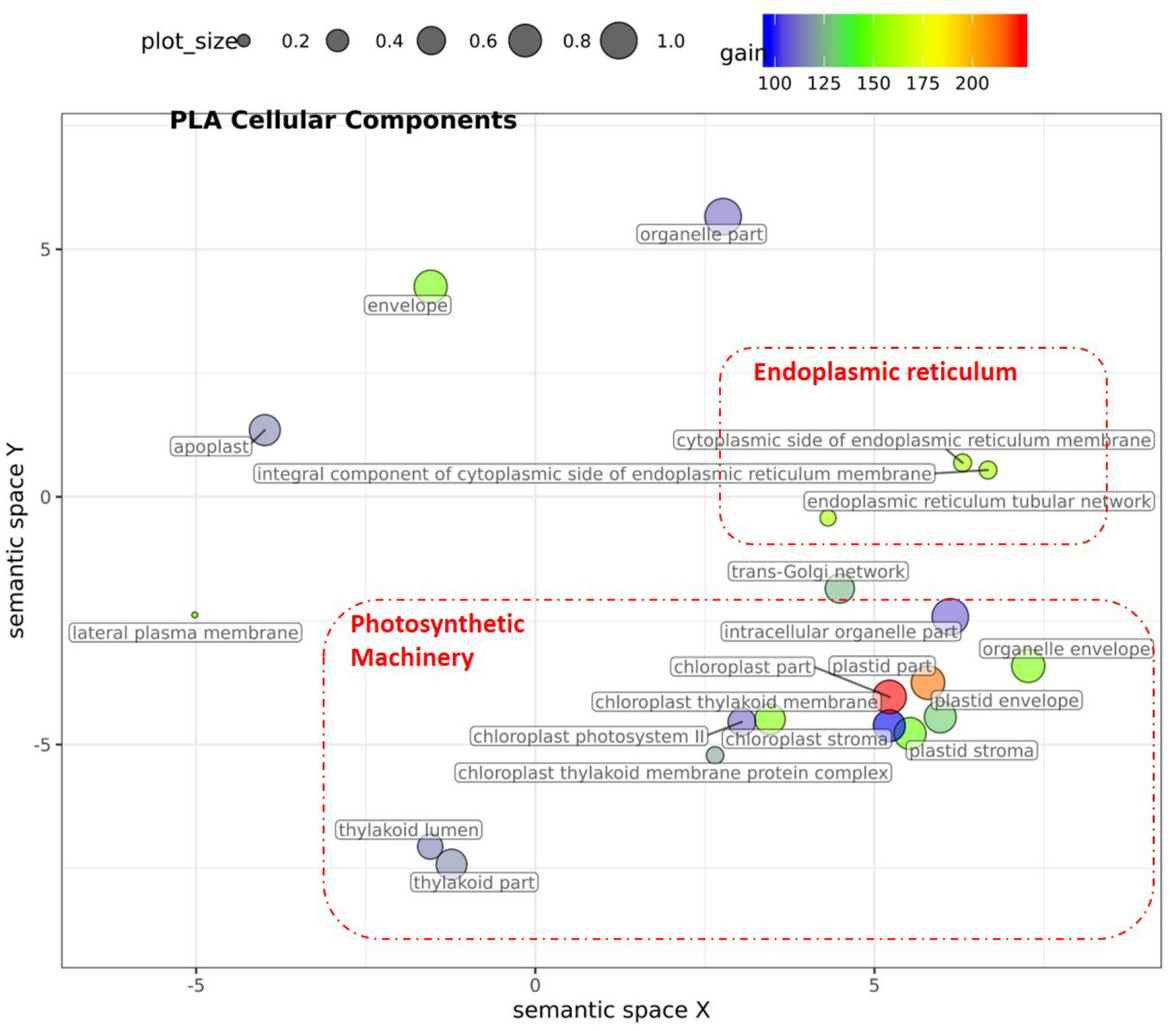
Semantic clustering of GO informed prediction for PLA. Multidimensional scaling (MDS) plot of the representative subset (i.e., terms remaining after the redundancy reduction) of cellular component GO terms informative for PLA. Semantically similar GO terms are clustered based on the “*SimRel”* semantic similarity measure using *Revigo* (Supek et al., 2011). Dot size is proportional to the number of genes annotated with the GO term, such that more general GO terms have larger bubbles. The *x* and *y* coordinates indicate relative virtual cluster distances in 2 dimensions. The %gain of a particular GO term is indicated by the bubble color.

For PLA, 52 GO terms (BP:41, CC:6, MF:5) resulted in significant improvement (*p*_FDR_ < 0.05) in predictive ability (Figure 5, Supplementary Figure 6C, Supplementary Table 2B) and the gain in accuracy ranged between 104 and 197%. After removal of hierarchical redundancy, semantic grouping of the remaining 45 GO terms showed that they involved a number of growth and developmental processes. Biological process GO terms fell into ~8 clusters (Figure 6, Supplementary Figure 10) related to development, defense response, stress response, cell cycle regulation, metabolism, molecular biosynthesis, cellular component organization, and transport. The molecular function terms were clustered into two groups including exopeptidase and methyltransferase activities. The cellular component terms included the photosynthesis machinery (i.e., chloroplast) and endoplasmic reticulum. Comparison of average accuracy over multiple folds of GO models (Supplementary Figures 6A,C) indicate that many models performed better than GBLUP. Some of these passed our significance threshold (see model evaluation criteria, M&M) at a particular trait measurement but appeared to improve prediction performance for other measurement points as well.

The maximum number of genes annotated with the informative GO terms for Φ_*PSII*_ and significant GO terms for PLA were 1,358 and 1,245, respectively. These GO terms appeared at multiple levels of the GO hierarchical structures, including parent and child terms closely related to photosynthesis and growth (Table 1). Moreover, many genes were common with the pre-selected photosynthesis related *PSGENES*: 42 and 58% for Φ_*PSII*_ and PLA respectively, significantly more than what expected by chance ($p_{\chi_{df:1}^{2}}<0.05$). Total genomic heritability ($h_{GBLUP}^{2}$) was negatively correlated with predictive gain (*r*_Φ*PSII*_ = −0.77, *r*_PLA_= −0.5). The genomic heritability explained individually ($h_{f}^{2}$) by the informative GO terms ranged between 6 and 31% for Φ_*PSII*_ and between 3 and 43% for PLA (Supplementary Tables 2A,B). Interestingly, the markers associated with these GO terms constituted only 0.1–3.3% of the total markers for Φ_*PSII*_ and 0.005–2.8% for PLA. This indicates that to improve predictive ability, genomic variance can be decomposed based on biologically meaningful sets of genes scattered over the genome rather than lie in adjacent regions such as in the MultiBLUP analysis above. Moreover, $h_{f}^{2}$ is positively correlated with GO gene group size (*r*_Φ*PSII*_ = 0.87, *r*_PLA_ = 0.77) as well as with the likelihood ratio (*r*_Φ*PSII*_ = 0.60, *r*_PLA_= 0.65) of both trait models, indicating that incorporating meaningful prior subsets into the GFBLUP model improves goodness of fit.

**Table 1:** Known trait-specific GO terms improving genomic prediction performance for both traits.

**GO ID**	**Ontology**	**Type**	**h${}_{\text{f}}^{2}$**	**LR**	***p*-value (unadj)**	**#gene**	**#marker**	**%gain**	**Cor(G_**f**_,G_**r**_)**	**h${}_{\text{GBLUP}}^{2}$**
**Φ*_PSII_***										
GO: 0009543	chloroplast thylakoid lumen	CC	0.07	10.53	1.48 × 10^−2^	71	218	33	0.59	0.09
GO: 0031968	organelle outer membrane	CC	0.06	12.47	4.3 × 10^−3^	72	345	40	0.61	0.08
GO:0044429	mitochondrial part	CC	0.14	47.05	2.3 × 10^−3^	298	1069	38	0.81	0.09
GO:0005740	mitochondrial envelope	CC	0.13	8.43	2.7 × 10^−2^	255	914	25	0.79	0.12
**GO ID**	**Ontology**	**Type**	**h**${}_{\text{f}}^{2}$	**LR**	***p*****-value (adj)**	**#gene**	**#marker**	**%gain**	**Cor(G**_**f**_**,G**_**r**_**)**	**h**${}_{\text{GBLUP}}^{2}$
**PLA**										
GO:0044434	Chloroplast part	CC	0.32	101	5.26 × 10^−5^	1211	5658	178	0.94	0.07
GO:0009535	chloroplast thylakoid membrane	CC	0.14	10	4.9 × 10^−2^	322	1139	121	0.81	0.07
GO:0000911	cytokinesis by cell plate formation	BP	0.15	34	9.6 × 10^−3^	204	1465	134	0.81	0.07
GO:0010090	trichome morphogenesis	BP	0.04	30	8.3 × 10^−4^	31	65	154	0.40	0.06
GO:0010321	regulation of vegetative phase change	BP	0.14	18	4.9 × 10^−3^	425	1512	106	0.84	0.07
GO:0048366	leaf development	BP	0.10	48	1.96 × 10^−5^	99	487	187	0.62	0.06
GO:0090698	post-embryonic plant morphogenesis	BP	0.04	7	8.3 × 10^−7^	4	11	207	0.20	0.06

*The proportion of explained genomic heritability (h${}_{f}^{2}$) by a GO term, likelihood ratio (LR) between GFBLUP and GBLUP models, Wilcoxon–Mann–Whitney test p-value, total number of genes and markers, %gain in accuracy (r), correlation between genomic relationship matrices based on GO term markers (G_f_) and remaining markers (G_r_) and total genomic heritability (h${}_{GBLUP}^{2}$), for different trait specific GO terms that are common to both GO and COEX based analyses. For GO terms, the type is indicated—molecular function (MF), biological process (BP) and cellular component (CC)*.

From this we infer that GO-based prior knowledge can improve GP performance. The improvement is most prominent for traits with low heritability, where some of the GO terms appeared more frequently for PLA than Φ_*PSII*_ at multiple measurement times.

### COEX Informed Prediction

Similar to genomic features based on GO, we made subsets of markers based on COEX clusters by selecting the markers within the ORFs of genes which were part of a given COEX cluster. Similar to GO based models, COEX models with zero and with 100% variance explained were discarded (Supplementary Figure 5). In general, more COEX models pass our model evaluation threshold (Supplementary Figures 6B,D) and they have a higher likelihood ratio than GO based models. This could be due to the genic overlap between groups and the enrichment of multiple related GO terms within a group.

**Figure 7:**
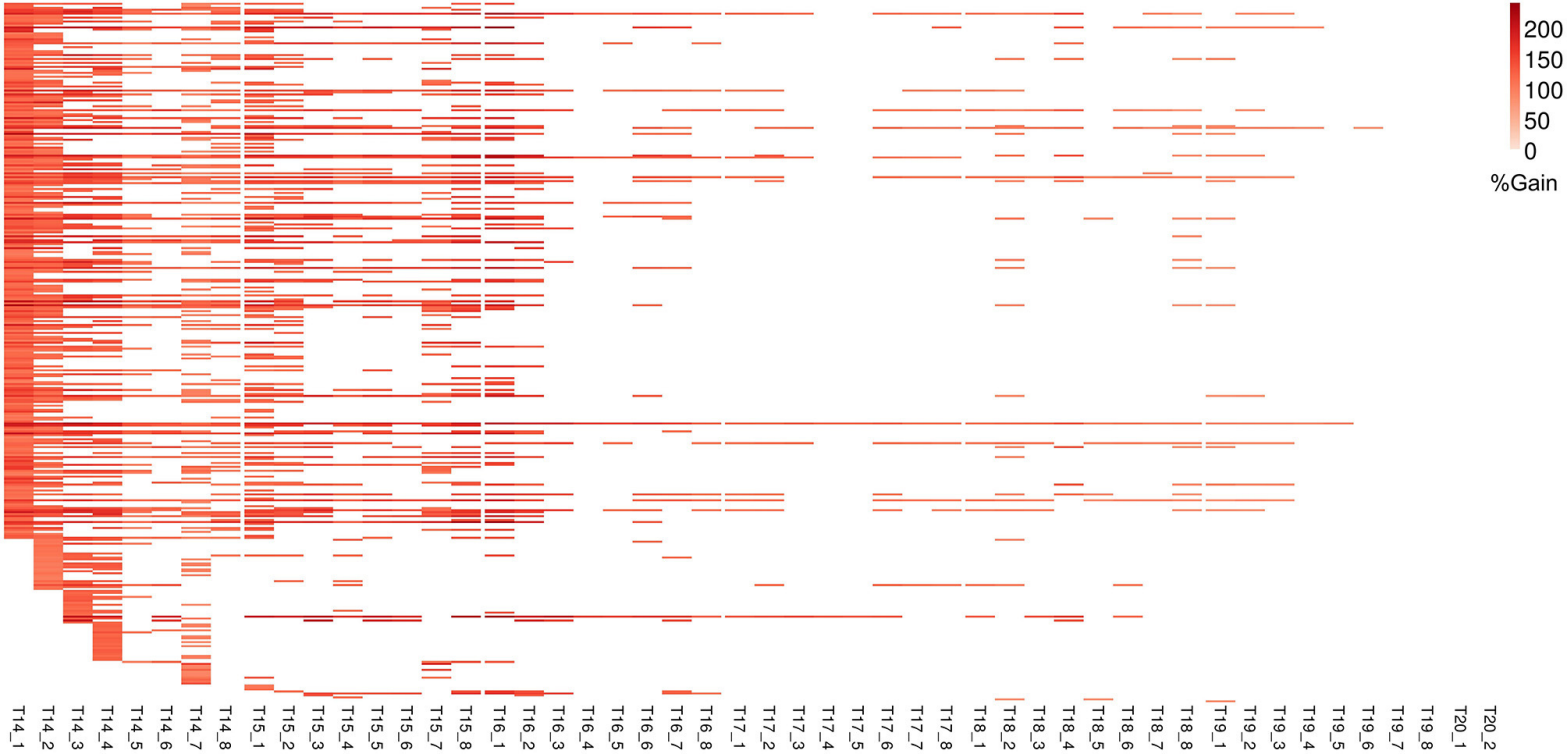
Improvement in genomic prediction performance using co-expressed gene clusters for PLA. All COEX clusters that significantly improve GFBLUP models for PLA with %gain in accuracy (*r*) over GBLUP. Each COEX cluster has a separate model for individual measurements indicated as T{day}_{Number of measurement}. The clusters are ordered according to “cluster_sr_no” column in Supplementary Table 3B.

For Φ_*PSII*_ we found 172 informative COEX gene groups potentially improving predictive ability, one of which was statistically significant (*p* < 0.05) after correcting for multiple testing using FDR (Supplementary Figures 6B, 8). 355 COEX groups significantly improved predictive ability for PLA (Figure 7, Supplementary Figure 6D, Supplementary Tables 3A,B). The gain in accuracy was higher for PLA (80 to 243%) than for Φ_*PSII*_ (7 to 89%) and was negatively correlated with genomic heritability (*r*_Φ*PSII*_ = −0.86, *r*_PLA_ = −0.56), like for GO informed prediction. This improvement was attributed to a maximum of only ~5% of the total genomic markers in all groups. Interpretation of COEX gene groups is not as straightforward as of GO terms, which by nature carry an informative name. Interestingly, ~90% of genes were common in the COEX groups for both traits, possibly due to the relatedness of the traits. To attach biological meaning to these groups we performed GO enrichment analysis on all groups together. We found 113 BP, 29 MF, and 24 CC most specific GO terms enriched in these clusters. The top 10 GO terms with highest fold enrichment include photosynthesis machinery, i.e., chloroplast stroma (GO:0009570), chloroplast envelope (GO:0009941) cellular components; ATPase activity coupled with transmembrane ion transport (GO:0015662); and glucose metabolic process (Supplementary Figure 11, Supplementary Table 5). These results indicate that trait-specific co-expressed gene functional groups can also help improve prediction performance and that these groups capture biologically relevant functions.

Similar to GO informed prediction, ~34% of COEX genes were common to the pre-selected photosynthesis related genes (*PSGENES*) for both traits, but here this is close to what we expect by chance. This indicates that, even though the COEX groups contain only a limited subset of all genes, they are not biased toward photosynthesis genes. The gain in predictive ability and explained genomic heritability ($h_{f}^{2}$) for Φ_*PSII*_ by the top COEX gene group was higher (89% resp. 14%) than those for the top GO feature (60% resp. 13%). Similarly, for PLA the top COEX gene group achieved a higher accuracy gain (242%) than the top GO group (197%), as shown in Figure 2. Notwithstanding these differences, we observed that many genes were common between GO and COEX based prediction for both traits (21 and 19% of all models passing the evaluation criteria for Φ_*PSII*_ and PLA resp.). These common genes in COEX based prediction were mainly enriched for many fundamental photosynthesis and growth related GO terms (Supplementary Tables 7A,B), e.g., light harvesting in photosystem I and photosynthetic electron transport in photosystem II (BP), chloroplast (CC), and ATP binding (MF).

The largest informative COEX groups for Φ_*PSII*_ and for PLA only differ slightly in sizes (3,176 and 2,840 genes, respectively), but on average, COEX groups were larger than the GO groups for both traits. The 95th percentile of genomic heritability explained individually by the COEX groups ($h_{f}^{2}$) was 70% for Φ_*PSII*_ and 39% for PLA, indicating that some Φ_*PSII*_ models could be over-estimated. Analogous to GO, $h_{f}^{2}$ was positively correlated with COEX gene group sizes (*r*_Φ*PSII*_ = 0.88, *r*_PLA_ = 0.40) and likelihood ratio (*r*_Φ*PSII*_ = 0.27, *r*_PLA_ = 0.22), indicating that incorporating meaningful prior subsets into the COEX model improved goodness of fit.

Together, our results illustrate that both of the meaningfully specific GO terms and more general COEX groups of genes with interrelated functions may improve GP predictive performance.

## Discussion

### Predicting Photosynthesis

In this work, we aimed at improving GP performance by exploiting publicly available biological knowledge to group genes in three different ways: using our knowledge about the trait, using the Gene Ontology and using co-expression. Instead of developing new methodology, we focused on using existing BLUP methods, widely used in animal and plant breeding, to explore new sources of biological prior knowledge, e.g., clusters of co-expressed genes. The GFBLUP methodology was initially proposed for *Drosophila melanogaster* using Gene Ontology data as biological prior knowledge (Edwards et al., 2016). We also investigated to what extent different traits benefit from and the use of prior knowledge. Our results support a strong influence of different trait genetic architectures, since performance improvement was more evident for leaf area phenotypes than for Φ_*PSII*_.

The approach can be generally applied to complex traits, but here we focused on photosynthesis and plant size. Besides serving as a case study, photosynthesis is also interesting in its own right, for two reasons. First, the genetic architecture of photosynthesis, though well-studied over the previous decades, is still poorly described in the quantitative genetic context (Van Rooijen et al., 2017). Secondly, it is an important target for improvement in crop breeding (Long et al., 2015). Modest improvements in photosynthesis efficiency by engineering photorespiratory pathways have demonstrated enormous yield gains (Kromdijk et al., 2016; South et al., 2019). The yield model of Monteith (Monteith, 1977) suggests that increased light use efficiency of photosystem II holds great potential to meet global food challenges by increasing the conversion efficiency of intercepted irradiance into biomass (ε_c_) (Van Bezouw et al., 2019). Another determinant of plant growth rate is leaf area growth, involving precise regulation of photosynthesis machinery and growth hormones such as auxin (Zhang et al., 2017). Leaf area measurements from fluorescence based non-destructive optical phenotyping systems, can be efficiently used to screen plants at different growth stages with varying levels of photosynthetic rates (Weraduwage et al., 2015). Therefore, improved GP models for these traits could have impact in future crop breeding.

Following Edwards et al. (2016), we studied accuracy on internal test sets within the HapMap population. Further work is needed for data-driven selection of the most relevant terms for prediction on external test sets. For example, a possible strategy may be to select the feature with highest genomic variance explained, or with lowest p-value in the LRT we described. Our results indicate that biological priors driven GP models can be used to rank groups of genes potentially associated to the trait of interest along with improving prediction performance. The GWAS conducted on the same HapMap population for photosynthetic light use efficiency of photosystem II identified that the *A. thaliana* “Yellow Seedling 1” gene is involved in photosynthesis acclimation response (Van Rooijen et al., 2017). This *YS1* gene is annotated with GO Cellular Component terms chloroplast, intracellular membrane-bounded organelle and mitochondrion and GO Biological Process terms thylakoid membrane organization and photosystem II assembly. Our results using GO and COEX GP (Table 1) clearly demonstrate that these GO terms were most prevalent to improve the prediction and explain a large amount of genomic heritability. This indicates that genomic prediction and GWAS support each other as potentially useful tools for forward genetics.

The gain of predictive accuracy of the GP models compared to the base-model is trait-specific and negatively correlates with genomic heritability, which is promising for breeding at low *h*^2^. This inverse relation may be due to the fact that we deal with highly polygenic, complex traits: many physiological and regulatory biological processes are involved in Φ_*PSII*_ under high light stress, e.g., PSII repair, ROX etc. Our models, testing groups of genes individually, may not be able to improve performance for such cases. Another potential explanation lies in the ability of GFBLUP to capture small genetic variance at low *h*^2^ in a separate random component, potentially including known causal genes, which is not possible in GBLUP.

### Exploiting Biological Knowledge to Improve Genomic Prediction

With recent technological advances in both field and controlled environment high-throughput phenotyping systems, phenotypes can be measured at unprecedented scales. Phenotypes can vary in space and time due to genetics and environment alone, genotype-by-environment (GxE) interactions as well as stochastic and development effects. Component variances due to these factors can be calculated by precise modeling. If multiple measurements are available, GP models can be developed on individual measurements, treated as individual phenotypes, or on derived parameters, e.g., growth curves. We found that at each measurement timepoint, at least some GO (in particular cellular component terms) or COEX group could help to improve performance, and some were more frequent (Figure 4, Supplementary Figure 7). For example, for Φ_*PSII*_ no single GO or COEX gene group was capable of improving GP accuracy for all time points (either LL or HL separately), but a number of gene groups were able to improve PLA at multiple measurements (although not always meeting the threshold for significance). Phenotyping at an extended scale and GP modeling thus provides an opportunity to obtain biological insights. As an alternative to modeling at each timepoint separately, a whole time series or growth curve can be used instead. We did not pursue this here, as time series data is not generally available in most practical scenarios and we were interested to learn whether performance improvement was specific to growth stages and conditions e.g., models for Φ_*PSII*_ behaved differently under low and high light conditions.

Here, we mainly investigated two approaches to incorporate publicly available trait-specific biological information into GP, i.e., pre-selecting a list of genes and selecting sets or groups of genes based on predicted functional (i.e., GO) or expression (COEX) information. The approach using predicted functional information proved to be more useful in this context, but more approaches and sources of information can also be incorporated with a focus on prioritizing biologically related genomic regions. Moreover, knowledge from multiple heterogeneous sources can be combined to further pinpoint potential QTLs, termed as poly-omics GP models (Wheeler et al., 2014; Uzunangelov et al., 2020). These information sources may include (i) predicted variants effects, (ii) gene functions e.g., GO, COEX, (iii) networks of gene-gene and protein-protein interactions, stored in public resources like STRING (Mering et al., 2003), GeneMANIA (Warde-Farley et al., 2010); (iv) pathways, in which genes are grouped e.g., KEGG (Kanehisa and Goto, 2000); (v) previously generated GWAS and QTL results which indicate involvement of particular regions for specific traits e.g., AraGWAS (Togninalli et al., 2020), AraQTL (Nijveen et al., 2017), (vi) known connections to phenotypes and (vii) endophenotypes, usually measured using -omics data at different stages of genetic information flow toward phenotypes. The reliability of these sources of information is an important factor for credible analysis. Information describing the (un)certainty of annotations is generally available in the form of a score (e.g., for gene functions based on GO evidence scores or reliability scores generated by a prediction method). It remains an open question how to incorporate such scores in the process of using the biological knowledge for GP.

Our first approach, pre-selecting a gene list, seems to be naive but can be useful as a baseline for comparison with more complex statistical procedures. The group based approach is usually based on gene function, but this heavily depends on computational prediction, as for most of the genes in plants and animals, no experimental function annotation is available (Radivojac et al., 2013). Function prediction is often based on sequence similarity, which works well for predicting molecular functions but less so for biological processes. Using expression compendia based on multiple experiments poses an interesting alternative, since genes with similar expression patterns are more likely functionally related, hence more likely involved in the same biological process(es) (Kourmpetis et al., 2011). Alternatives are to define phenotype associated genomic regions based on differential gene expression levels (Fang et al., 2017) or metabolite levels and metabolic fluxes (Tong et al., 2020), or to construct haplotypes in genic regions based on their ontology information (Gao et al., 2018). The GP requiring genomics inferred relationship matrices (GRM), e.g., GBLUP and its variants, can make use of information derived from these sources to construct a population variance-covariance structure (Zhang et al., 2010, 2011; Fragomeni et al., 2017). A simple approach is to include multiple random effects for each knowledge source yielding its own variance-covariance structure for the population under study, in the mixed model equations (Guo et al., 2016). One way to combine multiple omics datasets is to prepare a Composite Relationship Matrix (CRM) as a linear combination of Genomic Relationship Matrices (GRMs), Expression Relationship Matrices (XRMs), Metabolome Relationship Matrices (MRMs), MicroRNA Relationship Matrices (miRMs) etc. (Wheeler et al., 2014).

### Alternative Models for Genomic Prediction

Linear mixed model (LMM)-based genomic prediction, as used in this work, makes use of raw genotypes and parameter regularization to estimate thousands of SNP marker effects using only a few hundred observations (*p* >> *n*), employing different prior statistical assumptions on these parameters. This makes the approach fairly simple and interpretable; therefore, biological knowledge can be incorporated straightforwardly by employing these statistical assumptions. But with the increase in the ratio between markers and available phenotypes, serious overfitting problems may be encountered in these models (González-Recio et al., 2014), leading to a need to use prior knowledge in regularization. A more general set of statistical learning methods are Machine Learning (ML) methods for prediction and classification, capable of dealing with the dimensionality problem in a more flexible manner. In these methods, phenotypes are regressed on nonlinear functions of genotypes rather than raw genotype values, compromising model interpretability but potentially improving prediction performance. Several studies have reported the use of Support Vector Machines (SVM), Reproducing Kernel Hilbert Spaces Regression (RKHS), Neural Networks (NN), Random Forests (RF), and boosting (De Los Campos et al., 2010; Ogutu et al., 2011) for genomic prediction. Still, low prediction accuracy remains a problem for complex traits. It will be interesting to further explore how biological knowledge can be incorporated into ML approaches for GP. One way could be to involve a knowledge driven regularization-based approach as demonstrated for disease prediction in human (Deng and Runger, 2013).

## Conclusion

The wealth of publicly available transcriptomics and Gene Ontology based prior biological knowledge can be incorporated for genomic prediction of photosynthetic light use efficiency of photosystem II electron transport (Φ_*PSII*_) and PLA. Significant improvement in prediction accuracy over the benchmark GBLUP model was obtained for several GO terms and COEX groups. This improvement is trait-specific and negatively correlates with genomic heritability; whereas, for projected leaf area we found more added value than for Φ_*PSII*_. Many known photosynthesis-specific GO terms lead to improvements, providing evidence of the potential usefulness of this approach in future breeding practice. We foresee incorporation of heterogeneous prior biological information into machine learning algorithms as an active area of research in future.

## Materials and Methods

### Datasets

#### Genotype Data

Genotype data of the 360 natural accessions in the core set of the *Arabidopsis thaliana* HapMap population, representing its global diversity, was obtained using Affymetrix 250k SNP array (Zhang and Borevitz, 2009; Baxter et al., 2010). The HapMap accessions were chosen as most accessions are more or less equally interrelated, so modeling is not heavily affected by population structure. Phenotypes of 344 accessions were available, so 16 accessions were removed from the analysis (CS76104, CS76112, CS76254, CS76257, CS76121, CS28051, CS28108, CS28808, CS28631, CS76086, CS76138, CS76212, CS76196, CS76110, CS76117, CS76118). Genotype data were subjected to quality control and all genotypes with a missing call in any accession were removed. Only 510 (0.24%) markers had minor allele frequency (MAF) <0.01 and 14,824 (6.9%) had MAF <0.05 (Supplementary Figure 12). To incorporate the effects of rare alleles along with common alleles in the GP model, the MAF filtering threshold was set at 0.01. Of subsequent markers in a window of 50bp with a Pearson correlation coefficient (*r*) < 0.999, one was removed, using PLINKv1.9 (Purcell et al., 2007). In total, 214,051 SNPs passed quality filtering, 213,541 remained after MAF filtering and 207,981 SNPs were available after LD pruning for the analyses. The resulting minimal distance between SNPs was found to be ~550 bp.

#### Phenotype Data

The light use efficiency of Photosystem II electron transport (Φ_*PSII*_) dataset was obtained from Van Rooijen et al. (2017), who measured it using chlorophyll fluorescence via NIR imaging at 790 nm. In this dataset, Φ_*PSII*_ was recorded three times a day; under 100 μmol m^−2^ s^−1^ (low light) for 2 days and for four continuous days after induction of high light stress at 550 μmol m^−2^ s^−1^ to study the photosynthetic acclimatory response. We measured PLA every 3 h starting from the afternoon of day 22 after sowing until early morning of day 29 using the “*Phenovator”* high-throughput automated phenotyping system (Flood et al., 2016), which results in total of 54 timepoints for this trait (Supplementary Table 8). Technical mis-match errors between the imaging system and the coordination of image analysis software were identified for some replicates at some time points for a small number of genotypes, but these were not found to influence overall results and the data was thus retained. Data of timepoints on day 22 was excluded from the analyses due to their relatively low coefficient of variation.

The *Phenovator* system has been designed to screen Arabidopsis plants for photosynthesis and growth on a larger temporal scale in a carefully controlled environment with minimal noise. The plants are grown over a table, spatially arranged into sowing blocks, imaged using a moveable monochrome camera recording 12 plants per image, and processed using an image processing software (available on demand from the authors). The system design allows spatial uniformity and temporal reproducibility by minimizing the design parameter variances. Therefore, we expected low variances of interactions between genotype and the design parameters; whereas, within image position and sowing position could have larger main effects and thus could be corrected for. Phenotypic values were taken as the average of one to four replicates of Best Linear Unbiased Estimators (BLUE) using the linear mixed model adjusted for experimental design factors (Supplementary Table 9) that were described in Flood et al. (2016). For this experiment, the important design factors are spatial row (*x*) and column (*y*) coordinate, the image position and the sowing block. Thus, the BLUE for phenotypic mean is calculated based on this equation, implemented in R with the *lmer* function (supplemental R script) using the lme4 package (Bates et al., 2007):

(1) $$\begin{array}{l} Y=Genotype+x+y+Image\_position\\ \;+Sowing\_block+error \end{array}$$

where *Genotype* is used as fixed effect and the other factors are defined as random effects.

Both traits, at all measurement times, showed approximately normal distributions (Supplementary Figures 13, 14). The distributions are leptokurtic and left skewed for both traits (except for a few measurements for PLA on day 14 and day 15). The coefficients of variation under low light conditions for Φ_*PSII*_ ranged from 1.95 to 2.30% and 2.92 to 7.58% under high light and 18.73 to 27.04% for PLA (Supplementary Table 1). Correlation between subsequent measurement times was high (*r* > 0.9) for both traits, except between measurements under low vs. high light conditions of Φ_*PSII*_; therefore, these were analyzed separately.

#### Biological Priors

Co-expressed gene groups were obtained from the Arabidopsis expression compendium by Movahedi et al. (2011). GO data was retrieved using the R package “org.At.tair.db” (Carlson, 2019b) and genes were annotated using “GO.db” (Carlson, 2019a) irrespective of evidence codes. The set of genes in GO terms were up-propagated along the GO tree, such that each GO group in our analysis comprised of a set of all those genes attributed to itself or to all of its child terms. The up-propagated sets of genes were retrieved using the “GO2ALLTAIRS” method in the “org.At.tair.db” package. Markers in genes linked to a specific GO term or COEX cluster were used in the analyses.

Moreover, a set of 7,242 photosynthesis related genes was manually compiled (Supplementary Table 6) using four publicly available sources: KEGG (Kanehisa, 2001) pathways related to photosynthesis (i.e., ath00195, ath00197, ath00710); the Arabidopsis pathway database AraCyc for four photosynthesis pathways (i.e., Calvin cycle, photorespiration, oxygenic, light reaction); genes annotated with GO terms directly related to photosynthesis machinery; and all 51 priority genes selected for GWAS of photosynthesis acclamatory response identified by for this HapMap population.

### Statistical Analysis

#### Linear Mixed Models

The Linear Mixed Model (LMM) with one random genomic component was used as baseline. This model (Equation 2), known as Genomic Best Linear Unbiased Prediction (GBLUP) (Habier et al., 2007; Vanraden, 2008) was used to predict marker effects, calculate genomic heritability ($h_{GBLUP}^{2}$) and the total additive genomic values, which is the sum of all marker effects:

(2) $$\begin{array}{r} \tilde{\text{y}}=\mu +\text{g}+\varepsilon \end{array}$$

Here, $\tilde{\text{y}}$ is an *n*x1 vector of adjusted phenotypes as described in section 5.1.2, **μ** is the overall mean, **g** is an *n*x1 vector of genomic values captured by all genomic markers such that $\text{g}=\hat{\text{g}}$ and **ε** is an *n*-vector of residuals. The random genomic values **g** and residuals were assumed to be independent, normally distributed as $\text{g}\sim N\left(0,\text{G}\sigma_{g}^{2}\right)$, $\text{ε}\sim N\left(0,\text{I}\sigma_{e}^{2}\right)$. Here **G** is the genomic relationship matrix (GRM), providing variance-covariance structure of genotypes calculated from all genomic markers and **I** is the identity matrix.

Accordingly, for each GO and COEX gene groups, another linear mixed model similar to GBLUP but with two random genomic components (Equation 3), known as Genomic Feature Best Linear Unbiased Predictor (GFBLUP) (Edwards et al., 2016) was applied:

(3) $$\begin{array}{r} \tilde{\text{y}}=\mu +\text{f}+\text{r}+\varepsilon \end{array}$$

This model differs from GBLUP in that the total estimated genomic value $\left(\hat{\text{g}}=\text{f}+\hat{\text{r}}\right)$ is partitioned into genomic value captured by markers in a GO/COEX group (**f**) and by the remaining markers ($\hat{\text{r}}$), such that $\text{f}\sim N\left(0,{\text{G}}_{\text{f}}\sigma_{f}^{2}\right)$, $\text{r}\sim N\left(0,{\text{G}}_{\text{r}}\sigma_{r}^{2}\right)$ and $\text{ε}\sim N\left(0,\text{I}\sigma_{e}^{2}\right)$. For both GBLUP and GFBLUP, total genomic value $\hat{\text{g}}$ of the test population was predicted conditional on observed phenotypes of the training population, using the approach mentioned by Edwards et al. (2016). The genomic relationship matrix **G** in the GBLUP model was constructed based on all genomic markers such that $G=\frac{WW^{\prime }}{m},$, where **W** is an *n*×*m* genotype matrix (*n* genotypes and *m* markers), centered and scaled such that its *i*^th^ column ${\text{w}}_{\text{i}}=\frac{\left({\text{z}}_{\text{i}}-2p_{i}\right)}{\sqrt{2p_{i}\left(1-p_{i}\right)}}$, where **z**_**i**_ is the *i*^th^ column vector of **Z** having minor allele counts (0, 1, or 2) as entries and *p*_*i*_ is the MAF of the *i*^th^ marker. In our case, all genotypic locations were homozygous, so genotypes are coded as 0 or 2. For the GFBLUP model, the genomic relationship matrix **G**_**f**_ for each GO or COEX group was calculated from the markers linked to that group; **G**_**r**_ was constructed from the remaining markers.

The MultiBLUP model (Equation 4) was constructed according to the Adaptive MultiBLUP strategy proposed by (Speed and Balding, 2014). Briefly, the total genome was divided into adjacent but 50% overlapping regions of 10 kb. The genomic markers within these regions were tested as a group to estimate their association with the phenotype (*p* < 10^−5^) and adjacent regions were merged if *p*_Bonferroni_ < 0.05. Subsequently, separate covariance matrices **K**_1_, **K**_2_,., **K**_*M*_ were constructed for each region (*M* regions in total) based on its markers and genomic values **g**_1_, **g**_2_,., **g**_*M*_ were estimated. The GRM based on all markers (equivalent to GBLUP) was used if no region was found significant. The total genomic value is $\hat{\text{g}}=\overset{M}{\underset{m=1}{\sum }}{\hat{\text{g}}}_{m}$ with i.i.d. ${\text{g}}_{m}\sim N\left(0,{\text{K}}_{m}\sigma_{m}^{2}\right)$ and $\text{ε}\sim N\left(0,\text{I}\sigma_{e}^{2}\right)$:

(4) $$\begin{array}{r} \tilde{\text{y}}=\mu +\sum_{m\;=\;1}^{M}{\text{g}}_{m}+\varepsilon \end{array}$$

Variance components in all of these LMMs were estimated using the average information restricted maximum-likelihood (REML) procedure (Johnson and Thompson, 1995) implemented in the *greml* method of the R package *qgg* (Rohde et al., 2020) for GBLUP/GFBLUP, using a maximum of 100 iterations at a tolerance level of 10^−5^; and LDAK v5.1 (http://dougspeed.com/) for MultiBLUP.

Total additive genomic value was predicted using 8-fold cross-validation. This involved training the model using 301 (78%) genotypes and using the remaining 43 for testing in each fold. The exact same accessions were used for both GBLUP and GFBLUP during each split to enable a fair comparison. Prediction accuracy of models was defined as Pearson correlation (*r*) between observed phenotypic values and predicted genomic values of the test population in each fold. The procedure was repeated 10 times, thus modeled predictive ability distributions consisted of 80 correlations or fewer if variances were over- or underestimated as described earlier by simulation studies (Kruijer et al., 2015). For comparison between models, the median of these correlations was used, and significance of the difference was tested using the non-parametric Wilcoxon–Mann–Whitney test for assessing significant differences in median accuracy between GBLUP and GFBLUP. Subsequently, *p*-values were adjusted for multiple-testing correction by calculating False Discovery Rate (FDR) based on total number of GO/COEX groups multiplied by total number of time points (Edwards et al., 2016). For Φ_*PSII*_ we also analyzed results without FDR adjustment, which are referred as “informative” as opposed to “significant” throughout the text.

### Model Performance Evaluation

GFBLUP models were compared to the benchmark GBLUP based on their goodness of fit, predictive ability and estimated genomic parameters. Using the likelihood ratio test (LRT) we tested the null-hypothesis $\sigma_{f}^{2}=0$. LRT *p*-values were based on the asymptotic distribution of the LRT-statistic, which is a mixture of a point mass at 0 and a χ^2^-distribution with 1 degree of freedom (d.o.f.) (Edwards et al., 2015). The significantly improved GFBLUP models (*p*_LRT_ < 0.05) having predictive abilities greater than the benchmark GBLUP (i.e., *p-value* of Wilcoxon-Mann-Whitney tests < 0.05) were filtered for subsequent analysis. Genomic parameters were calculated from variance estimates of both models to analyze only models passing the abovementioned filtering criteria. This includes total genomic heritability explained ($h_{GBLUP}^{2}=\frac{\sigma_{g}^{2}}{\left(\sigma_{g}^{2}+\sigma_{e}^{2}\right)}$) and proportion of genomic heritability explained by an individual GO/COEX group in GFBLUP models ($h_{f}^{2}=\frac{\sigma_{f}^{2}}{\left(\sigma_{f}^{2}+\sigma_{r}^{2}+\sigma_{e}^{2}\right)}$). In order to check if we obtained a higher number of *PSGENES* in GO/COEX groups than expected by chance, we used the chi-square test with 1 d.o.f. to compare the observed vs. expected frequencies of *PSGENES* in these groups.

### Semantic Clustering of GO Terms

Informative GO terms were clustered based on their semantic similarity using the *Revigo* (Supek et al., 2011) web server with “*SimRel”* semantic similarity metric equal to 0.7. The resulting GO clusters were plotted using a Multidimensional Scaling (MDS) plot in R, where maximum %gain in accuracy by each GO term was used to color the bubbles. GO terms enriched in COEX groups were found using the PANTHER classification system (Mi et al., 2019). Fisher's exact test was used for calculating enrichment *p*-values followed by multiple testing correction using the FDR, reporting enrichment at *p* < 0.05. These enriched GO terms were sorted in order of their GO hierarchical tree such that a child term was below its parent; thus, the most specific GO terms are the child GO terms in the bottom of that tree, were used for subsequent analysis.

## Data Availability Statement

All data and scripts have been uploaded to the Wageningen University & Research git server (https://git.wur.nl/faroo002/pub1).

## Author Contributions

MA and T-PN provided the genotype and phenotype datasets. MF performed the analyses. DR, AD, and HN were involved in designing the analyses and interpreting the results. WK helped with statistical analysis. MF wrote the manuscript with DR, AD, HN, and SM. All authors read the final manuscript.

## Conflict of Interest

The authors declare that the research was conducted in the absence of any commercial or financial relationships that could be construed as a potential conflict of interest.

## Supplementary Material

Supplementary Figure 1 Relation between genomic heritability and GBLUP predictive ability. GBLUP prediction accuracy is directly proportional to genomic heritability for both traits. **(A)** shows the relation between heritability and accuracy under low light (LL) and high light (HL) irradiance levels for Φ_*PSII*_, **(B)** shows the same for PLA.

Supplementary Figure 2 GBLUP accuracy (*r*) vs. genomic variance ($h_{GBLUP}^{2}$). Each dot corresponds to prediction accuracy (*r*) of GBLUP (*y*-axis) for each split of the data during cross-validation. The genomic variance explained by the model (*x*-axis) ranges from 0 to 1 and calculated as $h_{GBLUP}^{2}=\frac{\sigma_{g}^{2}}{\left(\sigma_{g}^{2}+\sigma_{e}^{2}\right)}$. Models at different measurement times are colored differently. **(A)** represents GBLUP models for Φ_*PSII*_ and contains two separate clouds of dots, representing LL (left) and HL (right) models with different heritability ranges. **(B)** represents GBLUP models for PLA.

Supplementary Figure 3 MultiBLUP predictive ability. The boxplots show the prediction accuracy (*r*) of MultiBLUP applied to 18 measurements of Φ_*PSII*_ and 50 measurements of PLA. The average accuracy is slightly lower than the average GBLUP accuracy (white star) for both traits. **(A)** shows the prediction accuracy under low light (LL) and high light (HL) irradiance levels for Φ_*PSII*_ whereas, **(B)** shows the same for PLA.

Supplementary Figure 4 Number of genes and markers in GO and COEX features. Total number of genes and markers associated with those genes for both types of genomic features, i.e., GO (left) and COEX (right).

Supplementary Figure 5 GFBLUP accuracy (*r*) vs. genomic variance ($h_{f}^{2}$) explained by a GO/COEX group. Each dot corresponds to prediction accuracy (*r*) of GFBLUP (y-axis) for each split of data during cross-validation for a particular GO **(A,C)** and COEX **(B,D)** group. The genomic variance explained by the particular GO/COEX (*x*-axis) ranges from 0 to 1. **(A,B)**: GFBLUP models for Φ_*PSII*_; **(C,D)**: GFBLUP models for PLA.

Supplementary Figure 6 GBLUP vs. GFBLUP predictive ability. Average prediction accuracy (*r*) of GBLUP vs. GFBLUP using GO terms **(A,C)** and COEX clusters **(B,D)** for Φ_*PSII*_ **(A,B)** and PLA **(C,D)**. The average was calculated over 80 splits of the data (8-fold cross-validation repeated 10 times), excluding models where variance was undetermined). Red dots indicate models that passed our model evaluation criteria (see M&M).

Supplementary Figure 7 Improvement in genomic prediction performance using informative GO terms for φ_PSII._ All informative GO terms with %gain in accuracy (*r*) of GFBLUP over GBLUP at multiple Φ_*PSII*_ measurement times, indicated by {Low|High light}{day}_{Number of measurement}. The color bar identifies GO terms as Biological Process (BP), Cellular Component (CC) or Molecular Function (MF).

Supplementary Figure 8 Improvement in genomic prediction performance using informative COEX groups for φ_*PSII*_. All informative COEX clusters with %gain in accuracy (*r*) of GFBLUP over GBLUP at multiple Φ_*PSII*_ measurement times, indicated by {Low|High light}{day}_{Number of measurement}.

Supplementary Figure 9 Semantic clustering of GO informed prediction for Φ_*PSII*_. Multidimensional scaling (MDS) plot of representative subset (i.e., terms remaining after the redundancy reduction) of informative GO terms molecular functions and cellular components, capable of improving predictive ability of GFBLUP models for Φ_*PSII*_. Semantically similar GO terms are clustered based on the “SimRel” semantic similarity measure using *Revigo*. Dot size is proportional to the number of genes annotated with a GO term in the TAIR9 reference genome annotation. The *x* and *y* coordinates indicate relative cluster distances in 2 dimensions. The %gain of a particular GO term is indicated by the bubble color.

Supplementary Figure 10 Semantic clustering of GO informed prediction for PLA. Multidimensional scaling (MDS) plot of representative subset (i.e., terms remaining after the redundancy reduction) of informative GO terms molecular functions and cellular components, capable of improving predictive ability of GFBLUP models for PLA. Semantically similar GO terms are clustered based on the “*SimRel*” semantic similarity measure using *Revigo*. Dot size is proportional to the number of genes annotated with a GO term in the TAIR9 reference genome annotation. The *x* and *y* coordinates indicate relative cluster distances in 2 dimensions. The %gain of a particular GO term is indicated by the bubble color.

Supplementary Figure 11 Top 10 enriched GO terms in COEX clusters for Φ_*PSII*_ and PLA. Top 10 most specific GO terms enriched in 172 informative COEX clusters for the Φ_*PSII*_ and 355 for PLA traits. The horizontal axis measures the fold enrichment, i.e., the observed fraction of genes annotated with a particular GO term divided by the expected fraction in the reference genome of *Arabidopsis thaliana*. Enrichment *p*-values were found using Fisher's exact test with multiple testing correction using False Discovery Rate (FDR); only terms with *p*_FDR_ < 0.05 are shown.

Supplementary Figure 12 Minor allele frequency spectrum (MAF). MAF distribution of all 214,051 chip markers. The orange bar represents all markers having MAF <5%, the red bar rare alleles with MAF <1%.

Supplementary Figure 13 φ_PSII_ phenotypic data distributions using Best Linear Unbiased Estimates (BLUE). Distributions of genotypic means of BLUE values of genotypes in the dataset.

Supplementary Figure 14 PLA phenotypic data distributions using Best Linear Unbiased Estimates (BLUE). Distributions of genotypic means of BLUE values of genotypes in the dataset.

Supplementary Table 1 Best Linear Unbiased Estimated Phenotypic data statistics.

Supplementary Table 2a Informative GO terms increasing GFBLUP prediction accuracy for Φ_*PSII*_.

Supplementary Table 2b GO terms significantly increasing GFBLUP prediction accuracy for PLA.

Supplementary Table 3a Informative COEX improving GFBLUP prediction accuracy for Φ_*PSII*_.

Supplementary Table 3b COEX significantly improving GFBLUP prediction accuracy for PLA.

Supplementary Table 4 Genomic features statistics.

Supplementary Table 5 Enriched Go terms in Φ_*PSII*_ and PLA COEX analysis.

Supplementary Table 6 List of genes used in GBLUP based on only photosynthesis genes markers.

Supplementary Table 7a GO Enrichment of common genes between GO and COEX based analysis for Φ_*PSII*_.

Supplementary Table 7b GO Enrichment of common genes between GO and COEX based analysis for PLA.

Supplementary Table 8 Raw measurements of Projected Leaf Area.

Supplementary Table 9 Average best linear unbiased estimates (BLUE) of Projected Leaf Area.
