# Supplementary figures and images for: Prior Biological Knowledge Improves Genomic Prediction of Growth-Related Traits in Arabidopsis thaliana
Source: Front Genet. 2021 Jan 20;11:609117. doi: 10.3389/fgene.2020.609117 (PMC7855462; doi:10.3389/fgene.2020.609117)

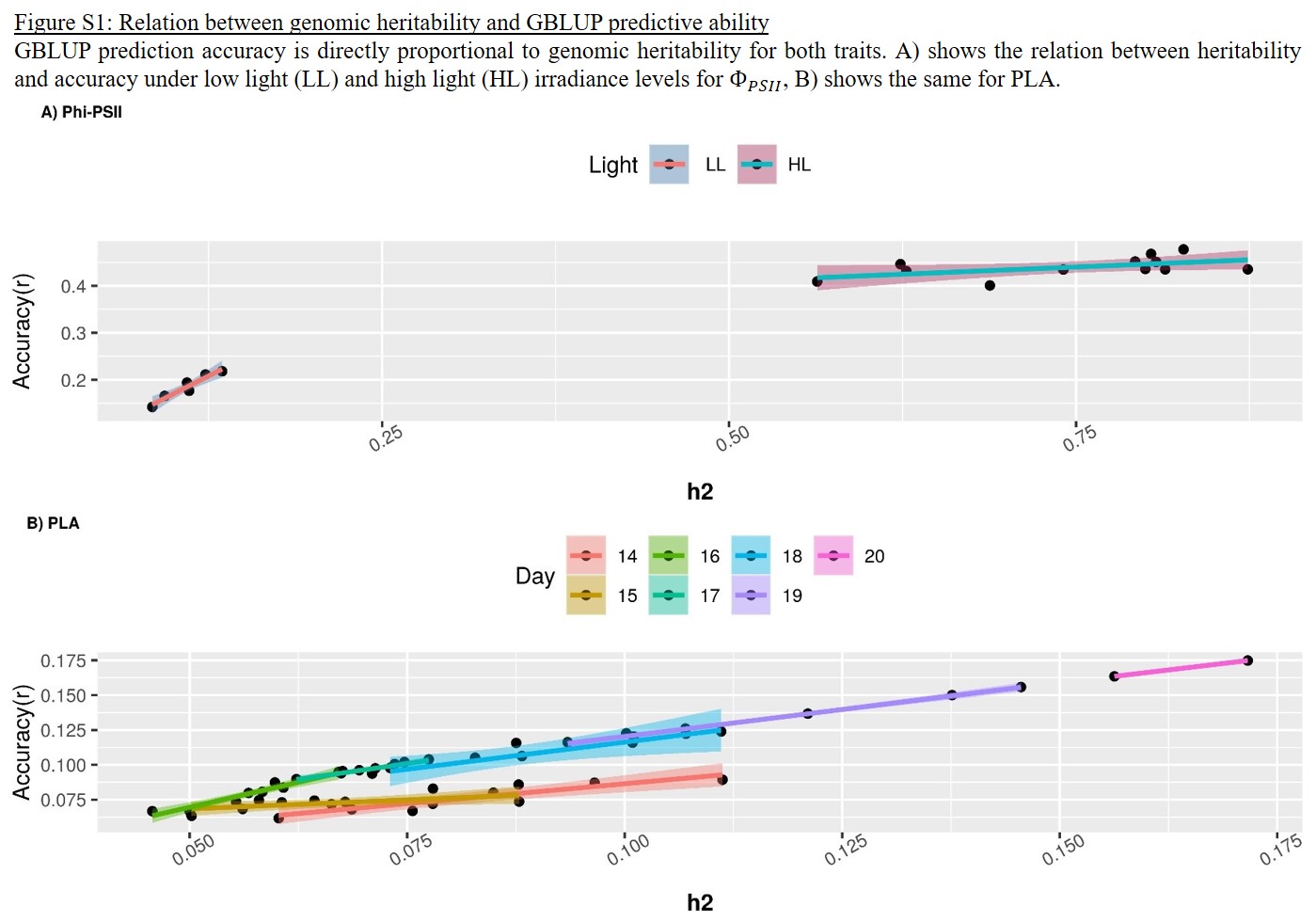

Supplement: Supplementary Figure 1 — Relation between genomic heritability and GBLUP predictive ability. GBLUP prediction accuracy is directly proportional to genomic heritability for both traits. (A) shows the relation between heritability and accuracy under low light (LL) and high light (HL) irradiance levels for ΦPSII, (B) shows the same for PLA. [file Image_1.JPEG]

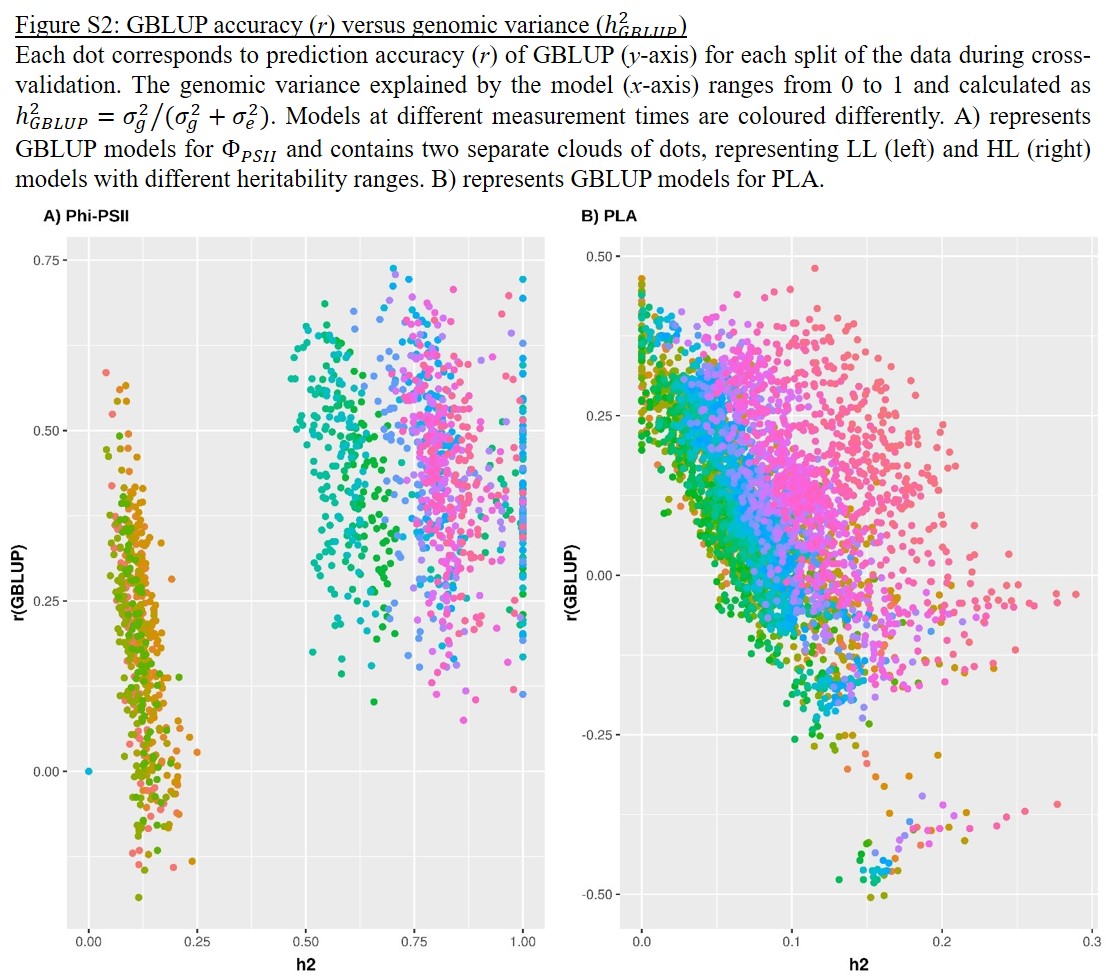

Supplement: Supplementary Figure 2 — GBLUP accuracy (r) vs. genomic variance (hGBLUP2). Each dot corresponds to prediction accuracy (r) of GBLUP (y-axis) for each split of the data during cross-validation. The genomic variance explained by the model (x-axis) ranges from 0 to 1 and calculated as hGBLUP2=σg2(σg2+σe2). Models at different measurement times are colored differently. (A) represents GBLUP models for ΦPSII and contains two separate clouds of dots, representing LL (left) and HL (right) models with different heritability ranges. (B) represents GBLUP models for PLA. [file Image_2.JPEG]

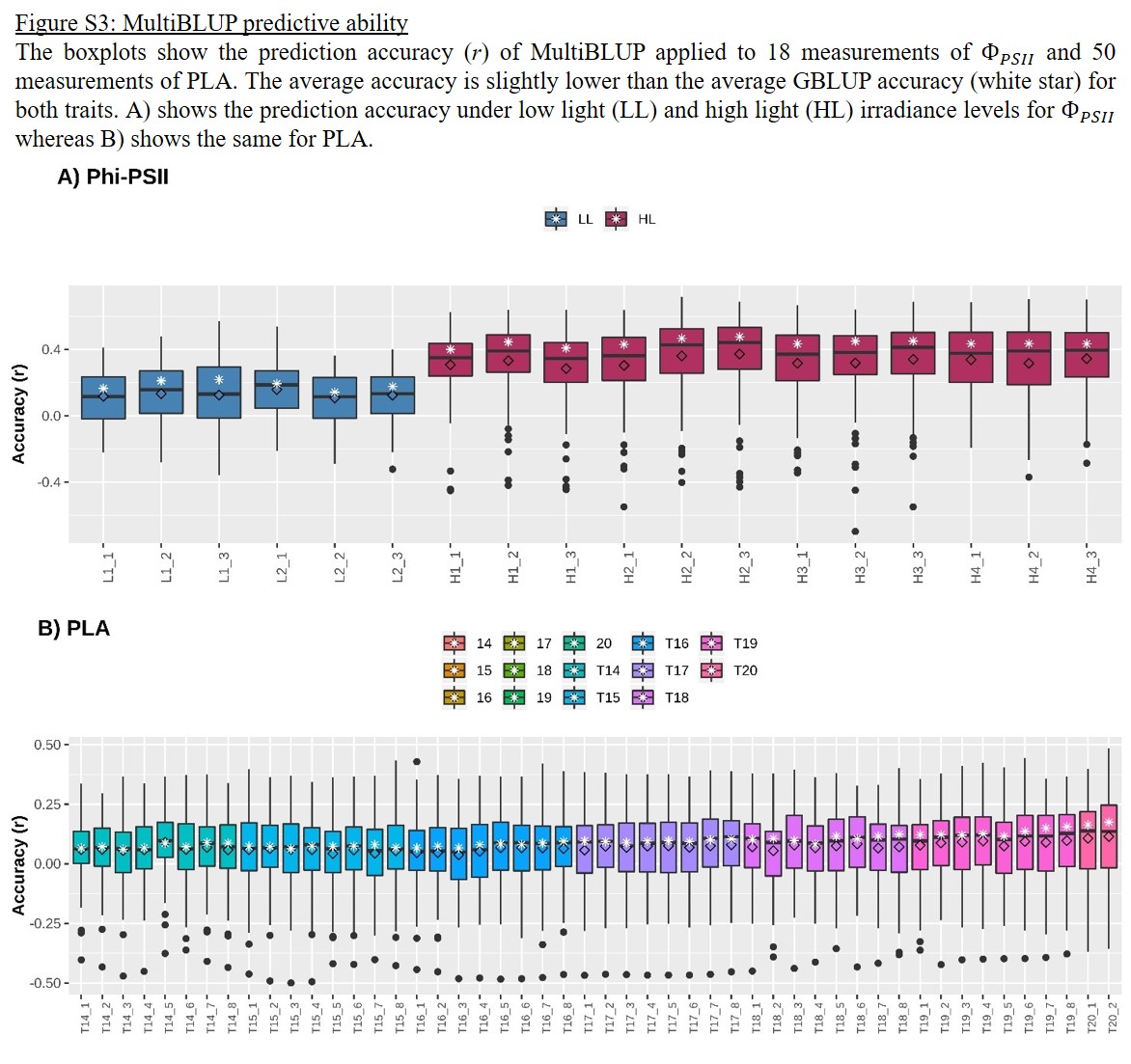

Supplement: Supplementary Figure 3 — MultiBLUP predictive ability. The boxplots show the prediction accuracy (r) of MultiBLUP applied to 18 measurements of ΦPSII and 50 measurements of PLA. The average accuracy is slightly lower than the average GBLUP accuracy (white star) for both traits. (A) shows the prediction accuracy under low light (LL) and high light (HL) irradiance levels for ΦPSII whereas, (B) shows the same for PLA. [file Image_3.JPEG]

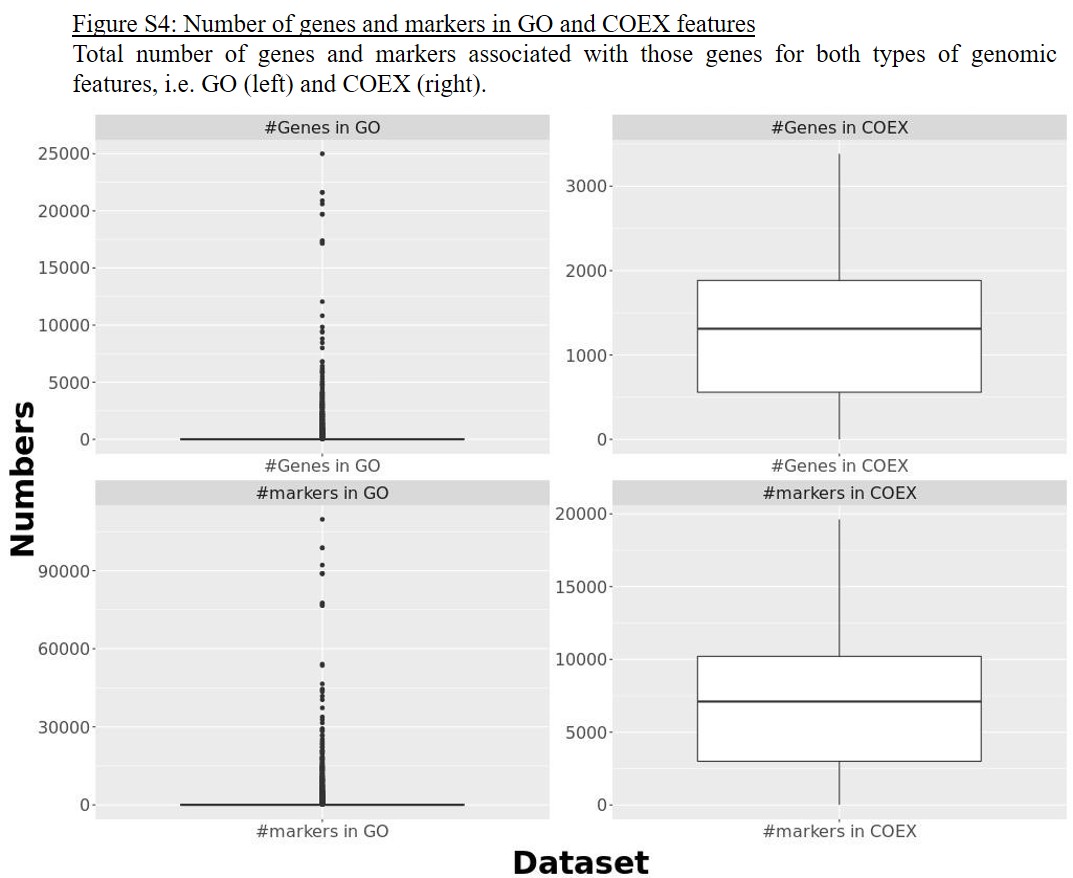

Supplement: Supplementary Figure 4 — Number of genes and markers in GO and COEX features. Total number of genes and markers associated with those genes for both types of genomic features, i.e., GO (left) and COEX (right). [file Image_4.JPEG]

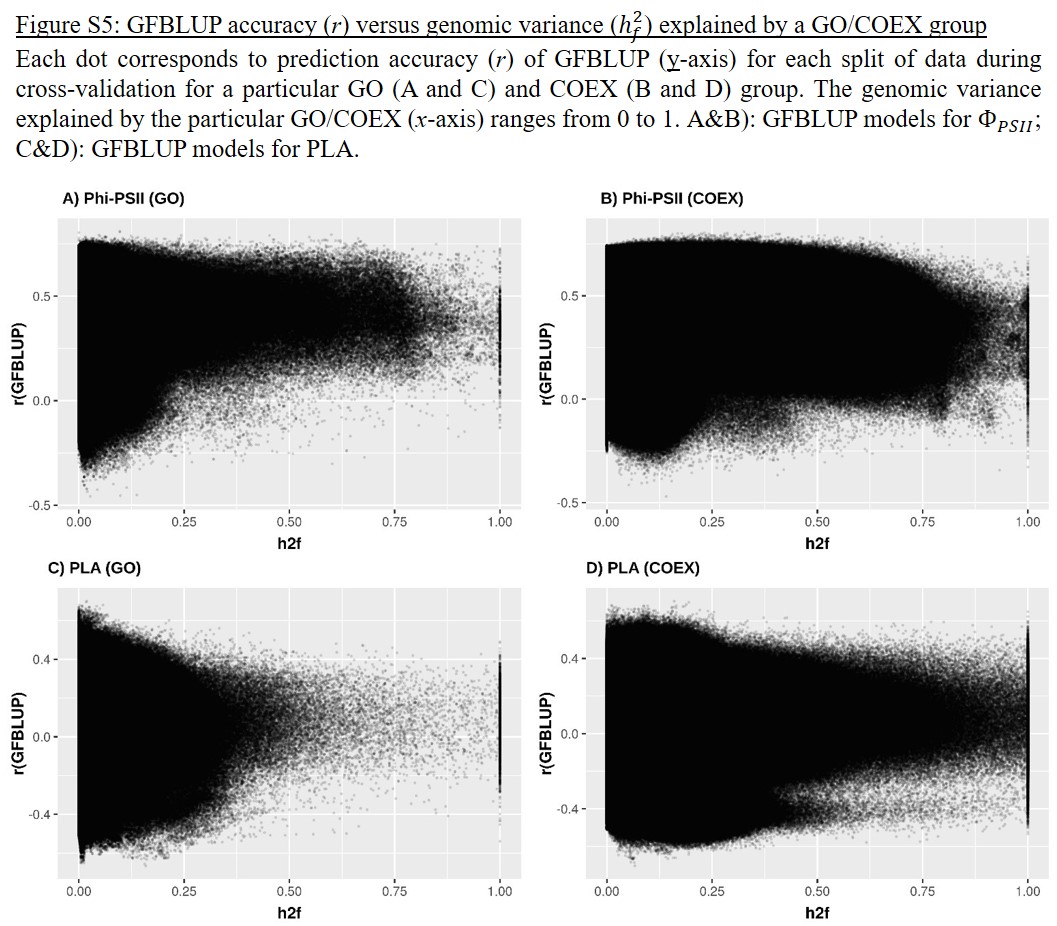

Supplement: Supplementary Figure 5 — GFBLUP accuracy (r) vs. genomic variance (hf2) explained by a GO/COEX group. Each dot corresponds to prediction accuracy (r) of GFBLUP (y-axis) for each split of data during cross-validation for a particular GO (A,C) and COEX (B,D) group. The genomic variance explained by the particular GO/COEX (x-axis) ranges from 0 to 1. (A,B): GFBLUP models for ΦPSII; (C,D): GFBLUP models for PLA. [file Image_5.JPEG]

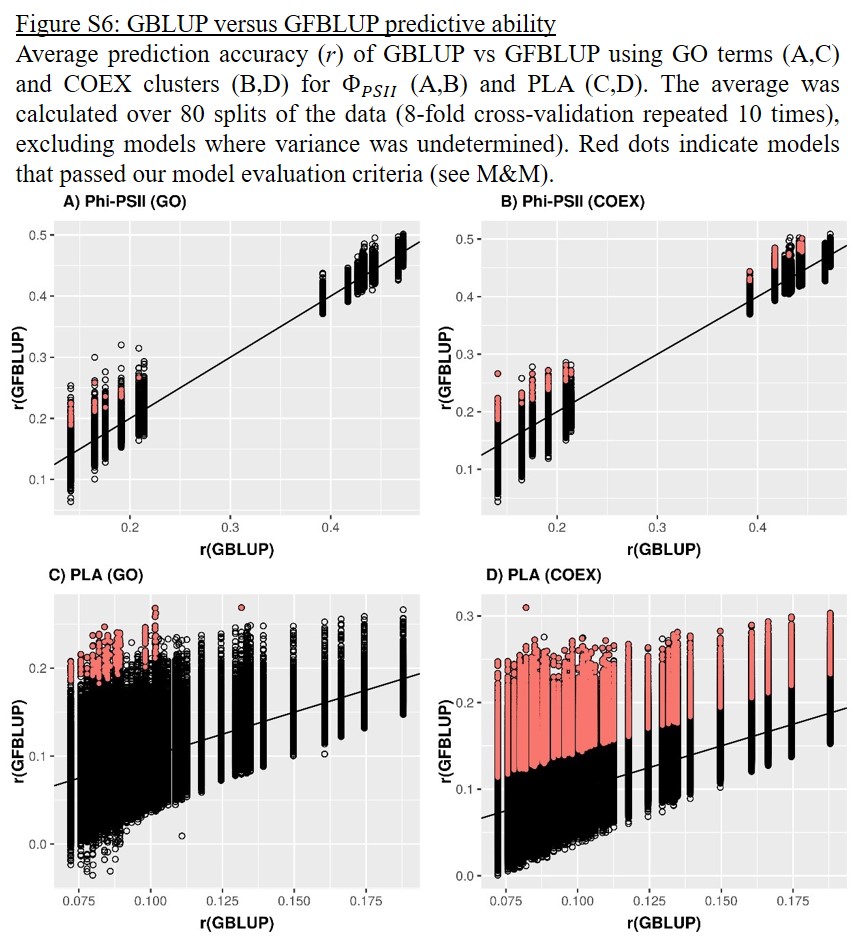

Supplement: Supplementary Figure 6 — GBLUP vs. GFBLUP predictive ability. Average prediction accuracy (r) of GBLUP vs. GFBLUP using GO terms (A,C) and COEX clusters (B,D) for ΦPSII (A,B) and PLA (C,D). The average was calculated over 80 splits of the data (8-fold cross-validation repeated 10 times), excluding models where variance was undetermined). Red dots indicate models that passed our model evaluation criteria (see M&M). [file Image_6.JPEG]

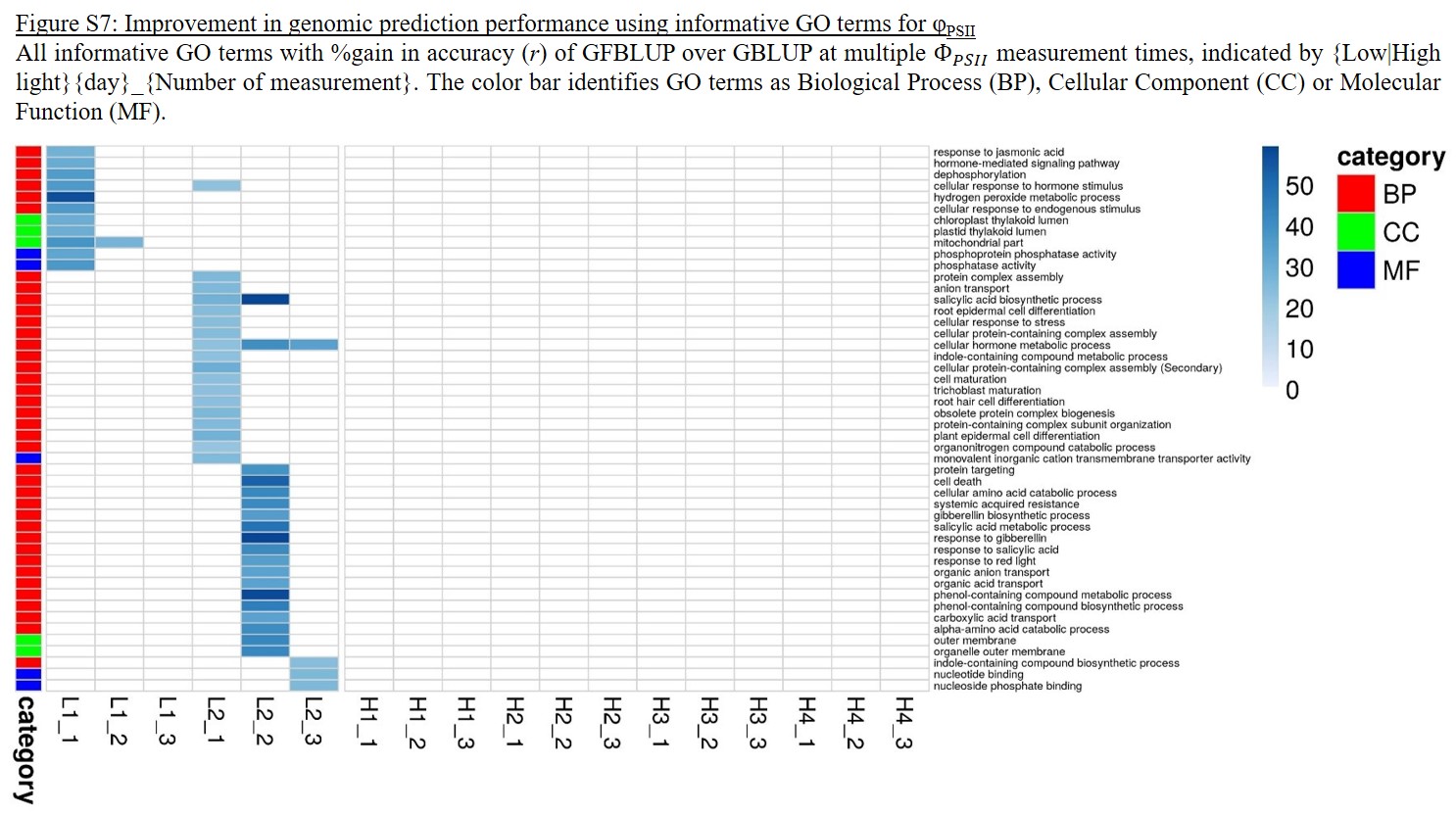

Supplement: Supplementary Figure 7 — Improvement in genomic prediction performance using informative GO terms for φPSII. All informative GO terms with %gain in accuracy (r) of GFBLUP over GBLUP at multiple ΦPSII measurement times, indicated by {Low|High light}{day}_{Number of measurement}. The color bar identifies GO terms as Biological Process (BP), Cellular Component (CC) or Molecular Function (MF). [file Image_7.JPEG]

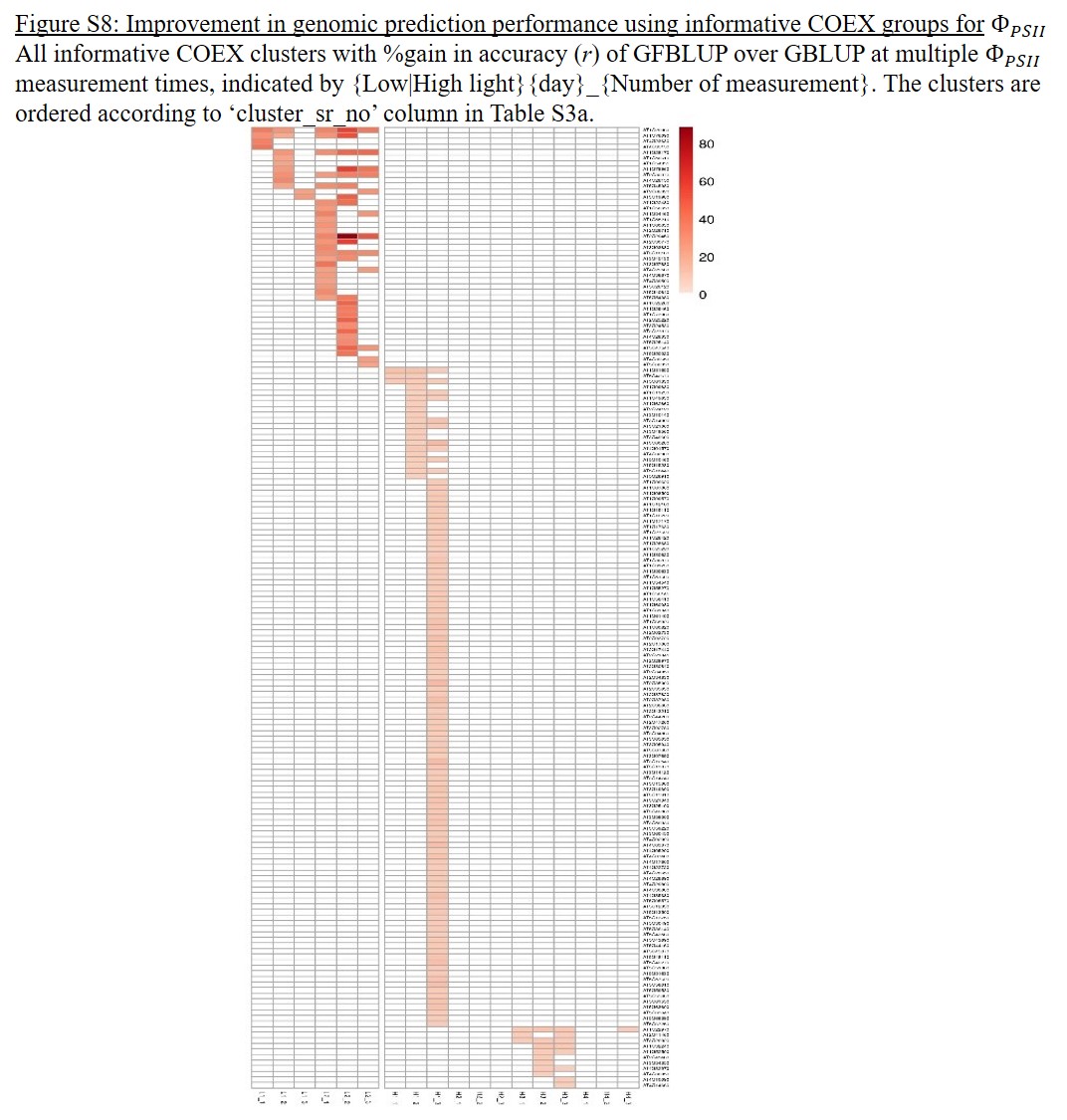

Supplement: Supplementary Figure 8 — Improvement in genomic prediction performance using informative COEX groups for φPSII. All informative COEX clusters with %gain in accuracy (r) of GFBLUP over GBLUP at multiple ΦPSII measurement times, indicated by {Low|High light}{day}_{Number of measurement}. [file Image_8.JPEG]

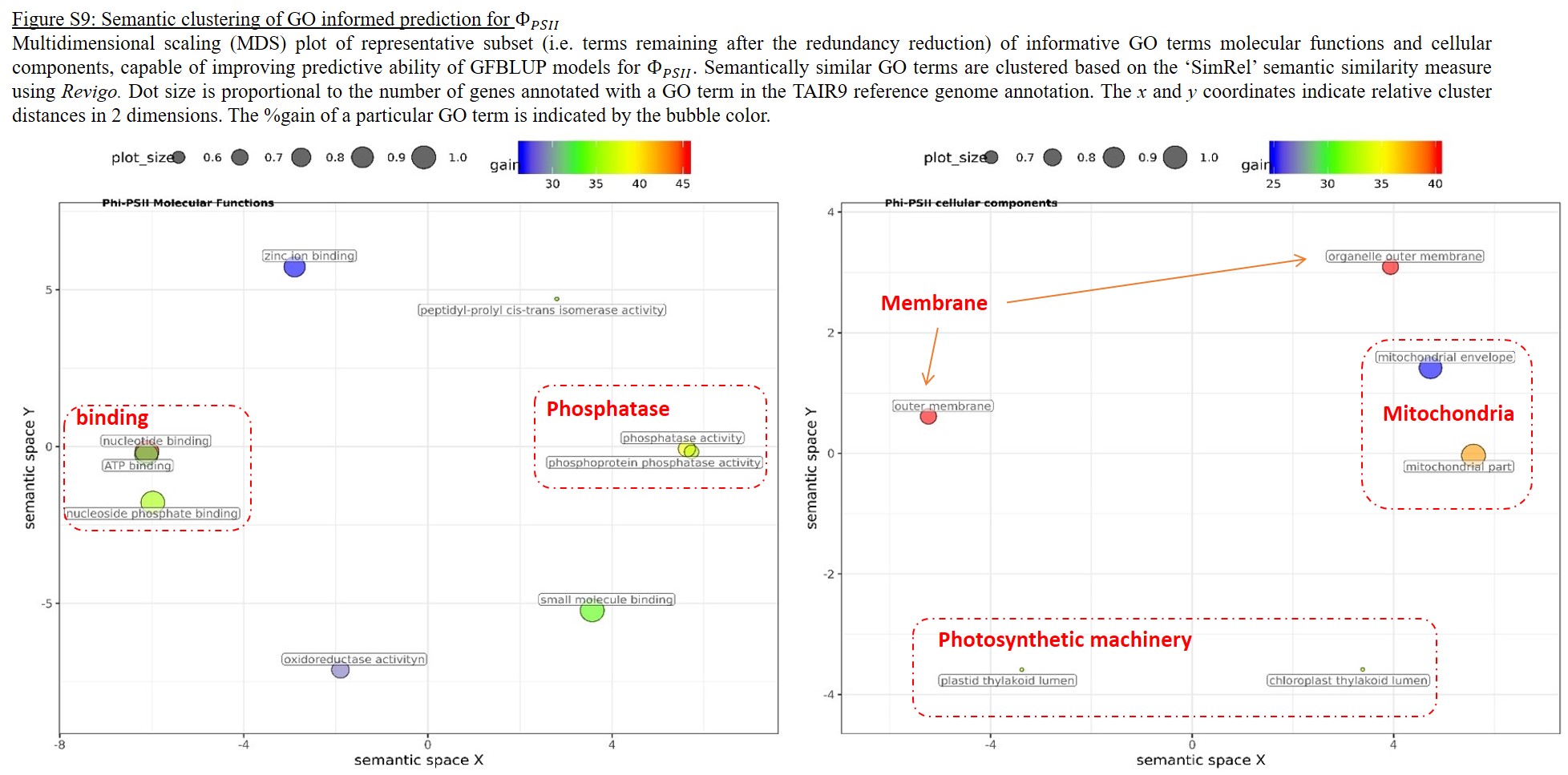

Supplement: Supplementary Figure 9 — Semantic clustering of GO informed prediction for ΦPSII. Multidimensional scaling (MDS) plot of representative subset (i.e., terms remaining after the redundancy reduction) of informative GO terms molecular functions and cellular components, capable of improving predictive ability of GFBLUP models for ΦPSII. Semantically similar GO terms are clustered based on the “SimRel” semantic similarity measure using Revigo. Dot size is proportional to the number of genes annotated with a GO term in the TAIR9 reference genome annotation. The x and y coordinates indicate relative cluster distances in 2 dimensions. The %gain of a particular GO term is indicated by the bubble color. [file Image_9.JPEG]

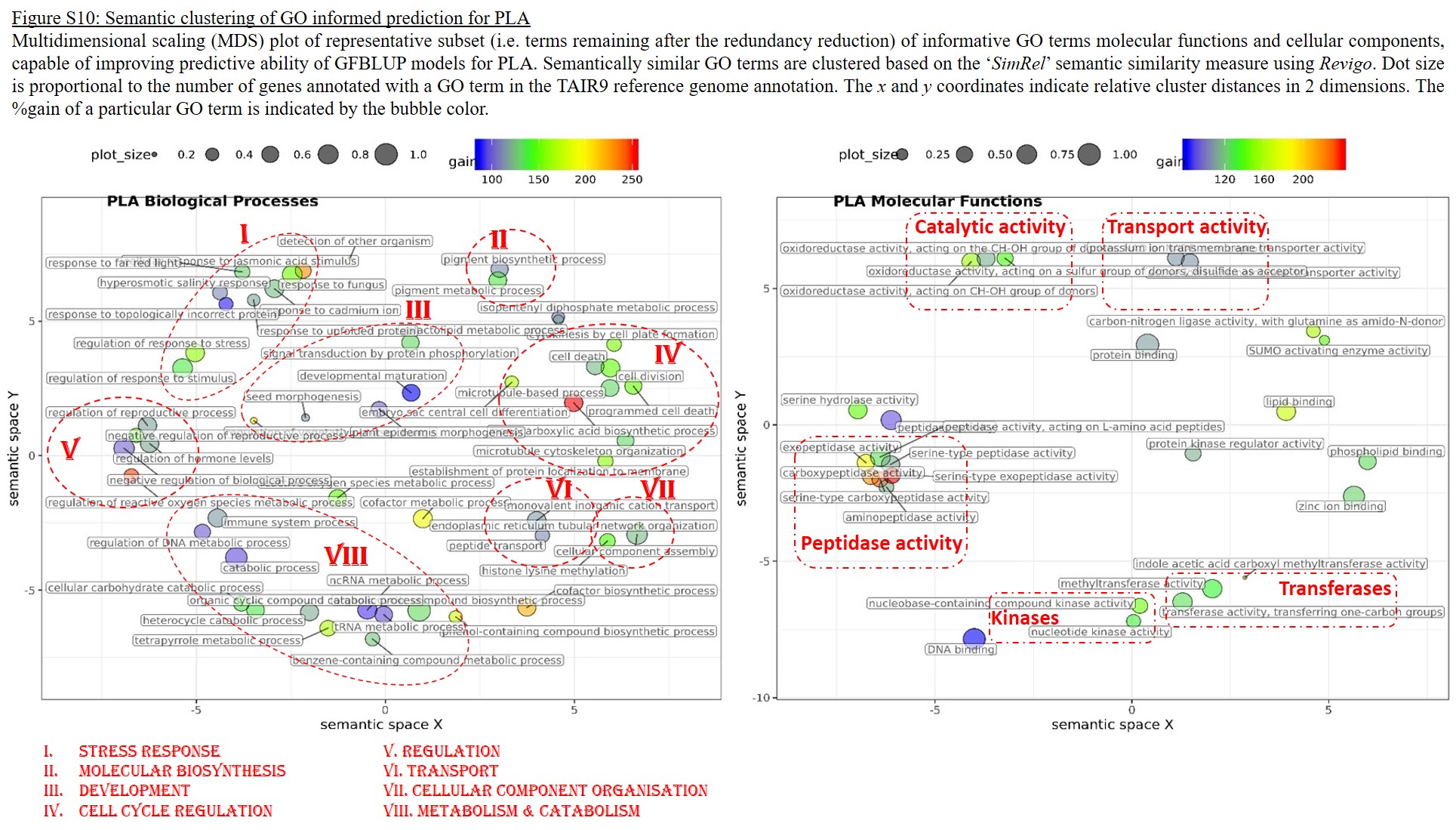

Supplement: Supplementary Figure 10 — Semantic clustering of GO informed prediction for PLA. Multidimensional scaling (MDS) plot of representative subset (i.e., terms remaining after the redundancy reduction) of informative GO terms molecular functions and cellular components, capable of improving predictive ability of GFBLUP models for PLA. Semantically similar GO terms are clustered based on the “SimRel” semantic similarity measure using Revigo. Dot size is proportional to the number of genes annotated with a GO term in the TAIR9 reference genome annotation. The x and y coordinates indicate relative cluster distances in 2 dimensions. The %gain of a particular GO term is indicated by the bubble color. [file Image_10.JPEG]

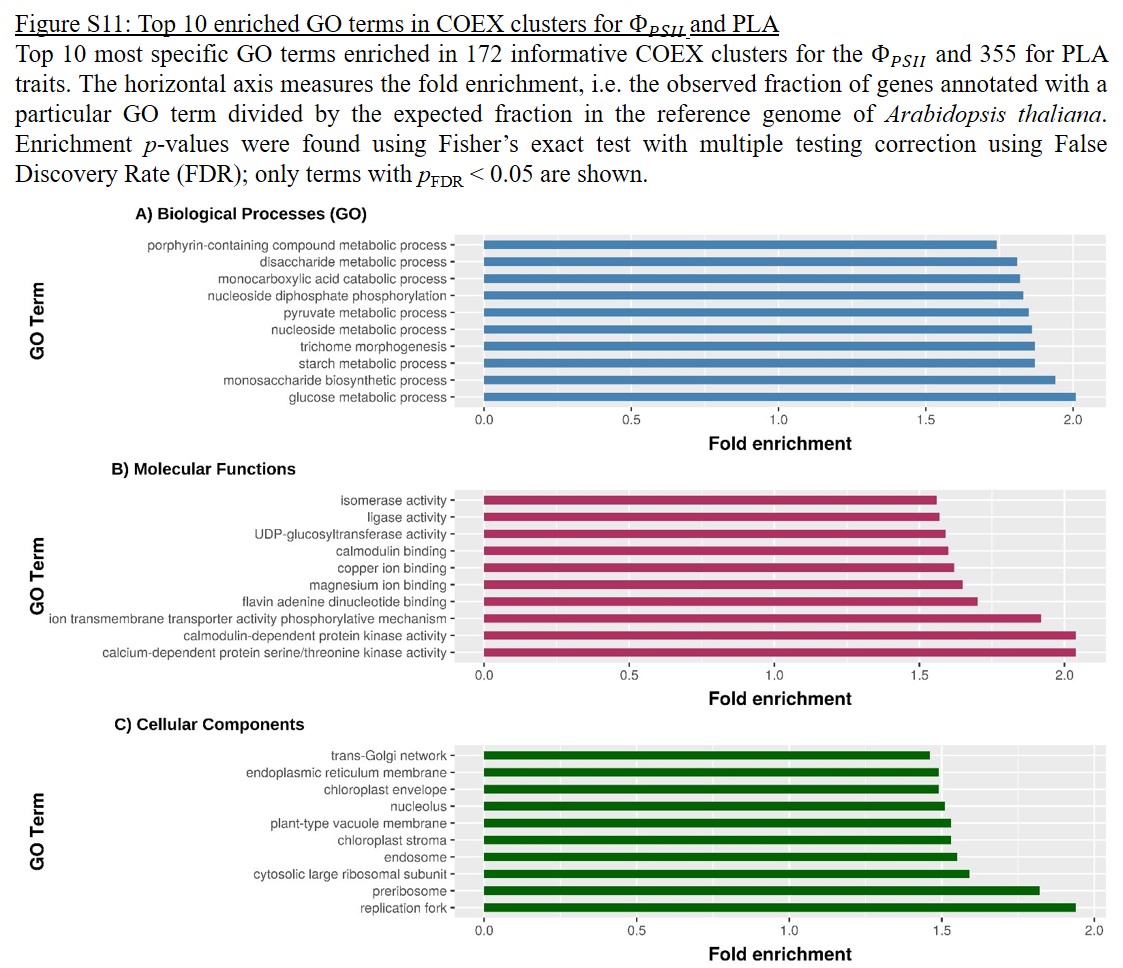

Supplement: Supplementary Figure 11 — Top 10 enriched GO terms in COEX clusters for ΦPSII and PLA. Top 10 most specific GO terms enriched in 172 informative COEX clusters for the ΦPSII and 355 for PLA traits. The horizontal axis measures the fold enrichment, i.e., the observed fraction of genes annotated with a particular GO term divided by the expected fraction in the reference genome of Arabidopsis thaliana. Enrichment p-values were found using Fisher's exact test with multiple testing correction using False Discovery Rate (FDR); only terms with pFDR < 0.05 are shown. [file Image_11.JPEG]

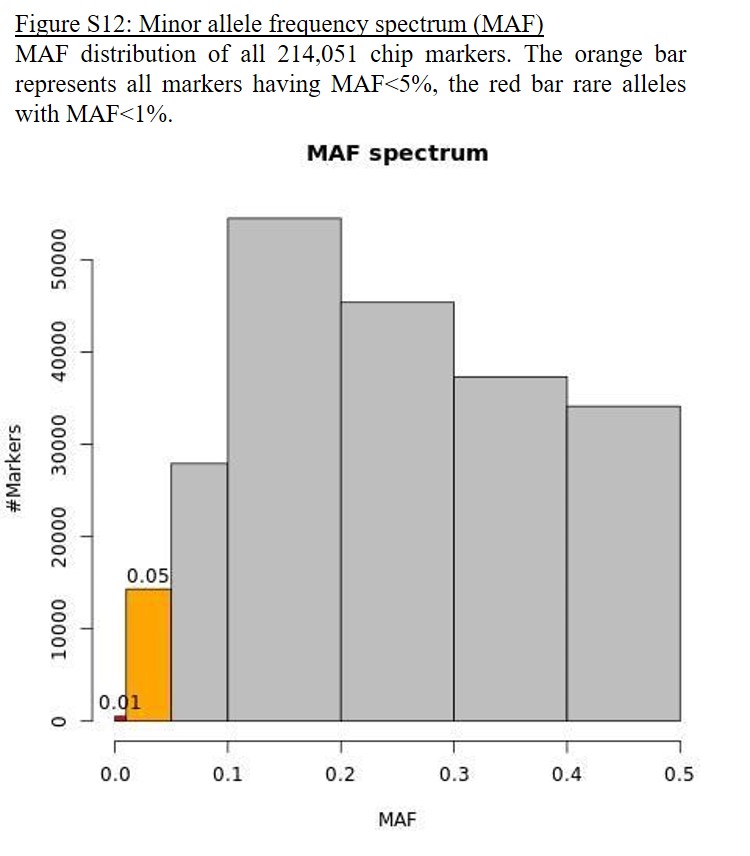

Supplement: Supplementary Figure 12 — Minor allele frequency spectrum (MAF). MAF distribution of all 214,051 chip markers. The orange bar represents all markers having MAF <5%, the red bar rare alleles with MAF <1%. [file Image_12.JPEG]

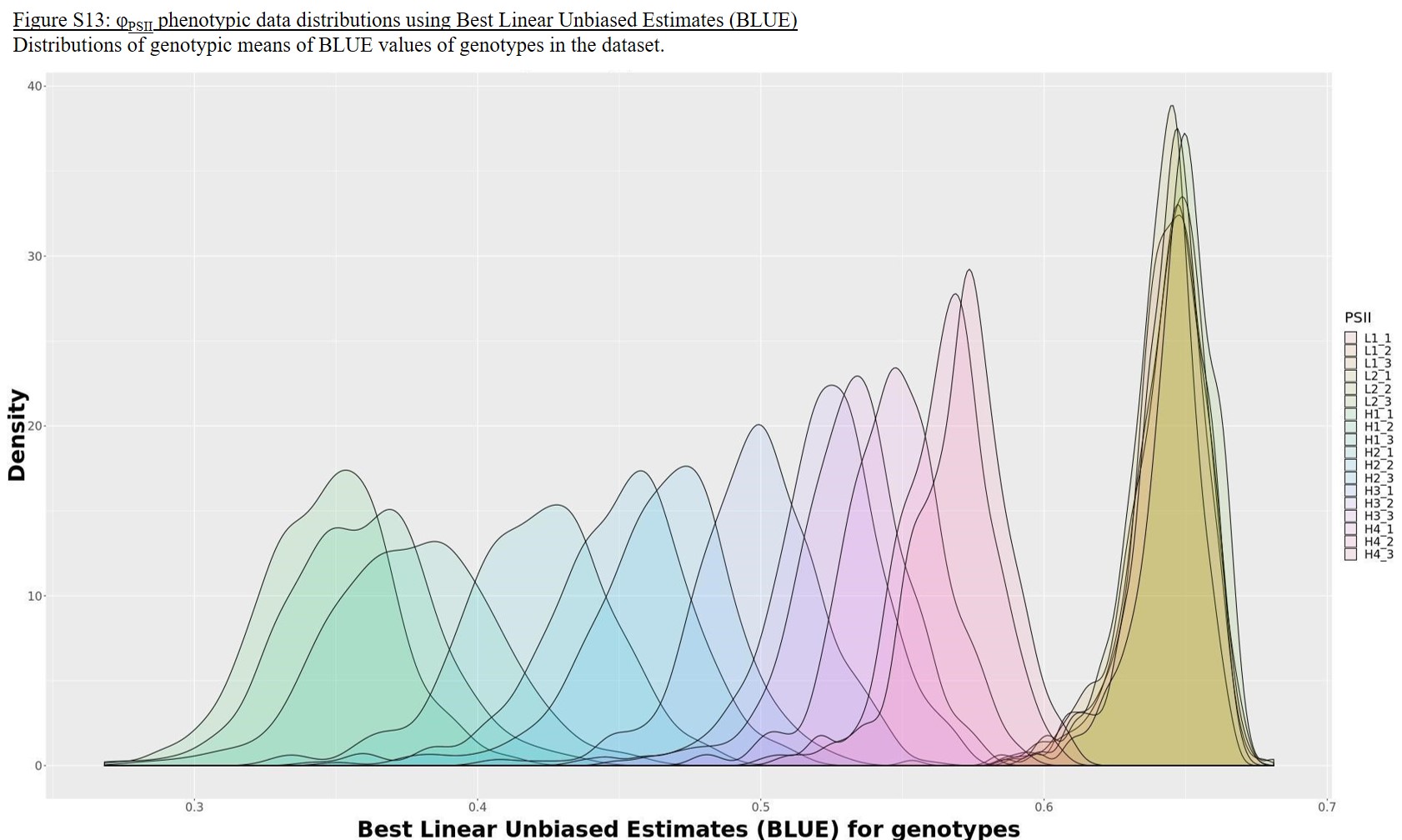

Supplement: Supplementary Figure 13 — φPSII phenotypic data distributions using Best Linear Unbiased Estimates (BLUE). Distributions of genotypic means of BLUE values of genotypes in the dataset. [file Image_13.JPEG]

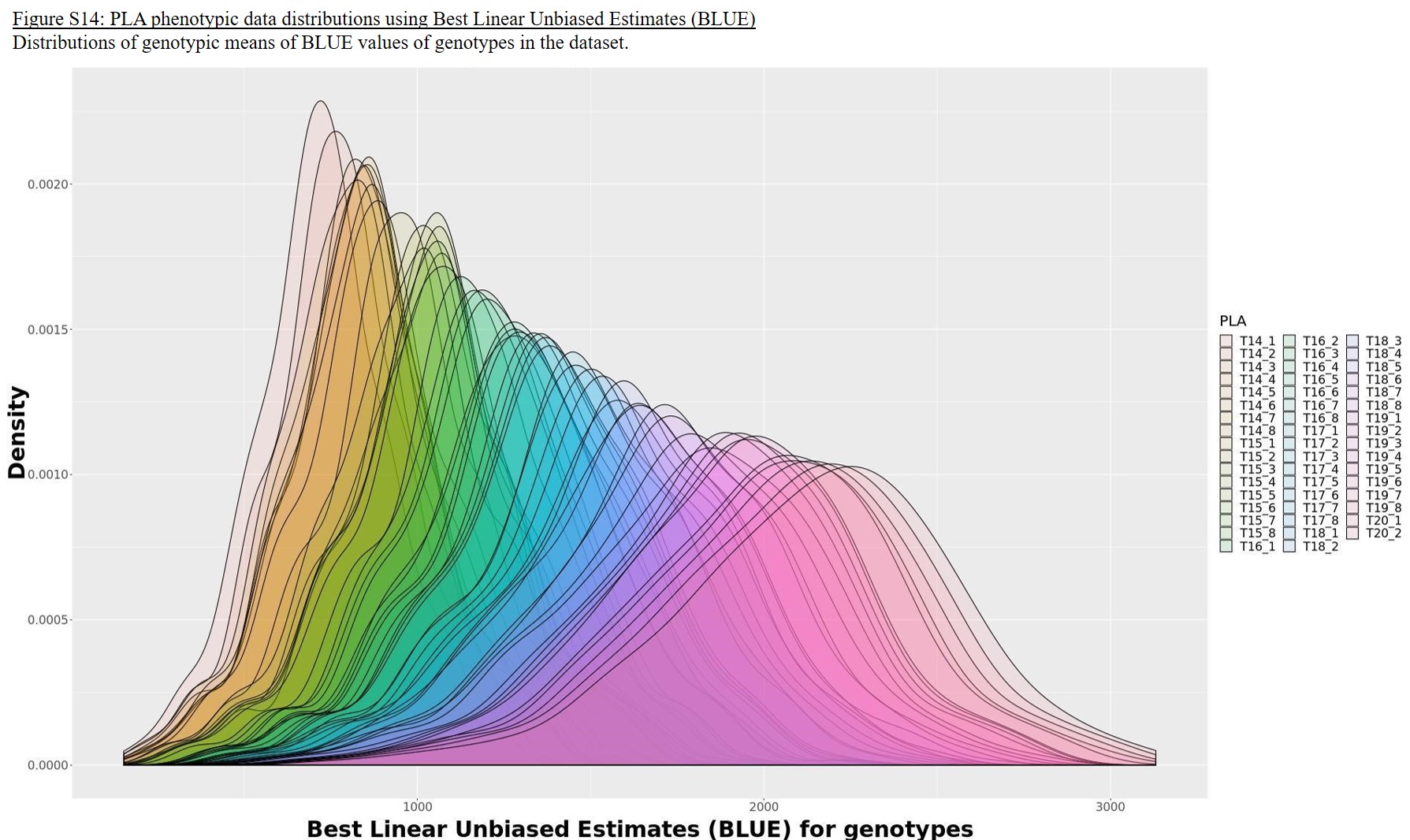

Supplement: Supplementary Figure 14 — PLA phenotypic data distributions using Best Linear Unbiased Estimates (BLUE). Distributions of genotypic means of BLUE values of genotypes in the dataset. [file Image_14.JPEG]
